# Supplementary material for: Co-Amorphous Drug Formulations in Numbers: Recent Advances in Co-Amorphous Drug Formulations with Focus on Co-Formability, Molar Ratio, Preparation Methods, Physical Stability, In Vitro and In Vivo Performance, and New Formulation Strategies
Source: Pharmaceutics. 2021 Mar 15;13(3):389. doi: 10.3390/pharmaceutics13030389 (PMC7999207; doi:10.3390/pharmaceutics13030389)
Supplement: Supplementary file 1 [file pharmaceutics-13-00389-s001.zip › Appendix B.pdf]

Appendix B. Studies included in this review article as the base for data analysis

Year 1989

Fukuoka E, Makita M, Yamamura S. Glassy state of pharmaceuticals. III: Thermal properties and stability of glassy pharmaceuticals and their binary glass systems. Chemical and Pharmaceutical Bulletin, 1989, 37: 1047-1050.

Year 1996

Yamamura S, Momose Y, Takahashi K, Nagatani S. Solid-state interaction between cimetidine and naproxen. Drug Stability, 1996, 1: 173-178.

Year 1998

Lu Q, Zografi G. Phase behavior of binary and ternary amorphous mixtures containing indomethacin, citric acid, and PVP. Pharmaceutical Research, 1998, 15: 1202-1206.

Year 2000

Yamamura S, Gotoh H, Sakamoto Y, et al. Physicochemical properties of amorphous precipitates of cimetidine–indomethacin binary system. European Journal of Pharmaceutics and Biopharmaceutics, 2000, 49: 259-265.

Year 2002

Yamamura S, Gotoh H, Sakamoto Y, et al. Physicochemical properties of amorphous salt of cimetidine and diflunisal system. International Journal of Pharmaceutics, 2002, 241: 213-221.

Year 2007

1. Hoppu P, Jouppila K, Rantanen J, et al. Characterisation of blends of paracetamol and citric acid. Journal of Pharmacy and Pharmacology, 2007, 59: 373-381.

2. Ahuja N, Katare O P, Singh B. Studies on dissolution enhancement and mathematical modeling of drug release of a poorly water-soluble drug using water-soluble carriers. European Journal of Pharmaceutics and Biopharmaceutics, 2007, 65: 26-38.

Year 2009

1. Allesø M, Chieng N, Rehder S, et al. Enhanced dissolution rate and synchronized release of drugs in binary systems through formulation: Amorphous naproxen–cimetidine mixtures prepared by mechanical activation. Journal of Controlled Release, 2009, 136: 45-53.

2. Chieng N, Aaltonen J, Saville D, et al. Physical characterization and stability of amorphous indomethacin and ranitidine hydrochloride binary systems prepared by mechanical activation. European Journal of Pharmaceutics and Biopharmaceutics, 2009, 71: 47-54.

3. Hoppu P, Virpioja J, Schantz S, et al. Characterization of ultrasound extrudated and cut citric acid/paracetamol blends[J]. Journal of Pharmaceutical Sciences, 2009, 98: 2140-2148.

4. Hoppu P, Virpioja J, Schantz S, et al. Characterization of ultrasound extrudated and cut citric acid/paracetamol blends[J]. Journal of Pharmaceutical Sciences, 2009, 98: 2140-2148.

5. Tantishaiyakul V, Songkro S, Suknuntha K, et al. Crystal structure transformations and dissolution studies of cimetidine–piroxicam coprecipitates and physical mixtures. AAPS Pharmscitech, 2009, 10: 789-795.

Year 2010

1. Tantishaiyakul V, Suknuntha K, Vao-Soongnern V. Characterization of cimetidine–piroxicam coprecipitate interaction using experimental studies and molecular dynamic simulations. AAPS Pharmscitech, 2010, 11: 952-958.

2. Pajula K, Taskinen M, Lehto V P, et al. Predicting the formation and stability of amorphous small molecule binary mixtures from computationally determined Flory− Huggins interaction parameter and phase diagram. Molecular Pharmaceutics, 2010, 7: 795-804.

Year 2011

Lȯbmann K, Laitinen R, Grohganz H, et al. Coamorphous drug systems: enhanced physical stability and dissolution rate of indomethacin and naproxen. Molecular Pharmaceutics, 2011, 8: 1919-1928.

Year 2012

1. Löbmann K, Strachan C, Grohganz H, et al. Co-amorphous simvastatin and glipizide combinations show improved physical stability without evidence of intermolecular interactions. European Journal of Pharmaceutics and Biopharmaceutics, 2012, 81: 159-169.

2. Masuda T, Yoshihashi Y, Yonemochi E, et al. Cocrystallization and amorphization induced by drug–excipient interaction improves the physical properties of acyclovir. International Journal of Pharmaceutics, 2012, 422: 160-169.

Year 2013

1. Gao Y, Liao J, Qi X, et al. Coamorphous repaglinide–saccharin with enhanced dissolution. International Journal of Pharmaceutics, 2013, 450: 290-295.

2. Löbmann K, Grohganz H, Laitinen R, et al. Amino acids as co-amorphous stabilizers for poorly water soluble drugs–Part 1: Preparation, stability and dissolution enhancement. European Journal of Pharmaceutics and Biopharmaceutics, 2013, 85: 873-881.

3. Löbmann K, Laitinen R, Strachan C, et al. Amino acids as co-amorphous stabilizers for poorly water-soluble drugs–Part 2: Molecular interactions. European Journal of Pharmaceutics and Biopharmaceutics, 2013, 85: 882-888.

4. Löbmann K, Laitinen R, Grohganz H, et al. A theoretical and spectroscopic study of co-amorphous naproxen and indomethacin. International Journal of Pharmaceutics, 2013, 453: 80-87.

5. Shayanfar A, Ghavimi H, Hamishekar H, et al. Coamorphous atorvastatin calcium to improve its physicochemical and pharmacokinetic properties. Journal of Pharmacy & Pharmaceutical Sciences, 2013, 16: 577-587.

6. Shayanfar A, Jouyban A. Drug–drug coamorphous systems: characterization and physicochemical properties of coamorphous atorvastatin with carvedilol and glibenclamide. Journal of Pharmaceutical Innovation, 2013, 8: 218-228.

7. Paluch K J, McCabe T, Muller-Bunz H, et al. Formation and physicochemical properties of crystalline and amorphous salts with different stoichiometries formed between ciprofloxacin and succinic acid. Molecular Pharmaceutics, 2013, 10: 3640-3654.

8. Shimada Y, Goto S, Uchiro H, et al. Features of heat-induced amorphous complex between indomethacin and lidocaine. Colloids and Surfaces B: Biointerfaces, 2013, 102: 590-596.

9. Shimada Y, Goto S, Uchiro H, et al. Characteristics of amorphous complex formed between indomethacin and lidocaine hydrochloride. Colloids and Surfaces B: Biointerfaces, 2013, 105: 98-105.

Year 2014

1. Dengale S J, Ranjan O P, Hussen S S, et al. Preparation and characterization of co-amorphous Ritonavir–Indomethacin systems by solvent evaporation technique: Improved dissolution behavior and physical stability without evidence of intermolecular interactions. European Journal of Pharmaceutical Sciences, 2014, 62: 57-64.

2. Jensen K T, Löbmann K, Rades T, et al. Improving co-amorphous drug formulations by the addition of the highly water soluble amino acid, proline. Pharmaceutics, 2014, 6: 416-435.

3. Laitinen R, Lobmann K, Grohganz H, et al. Amino acids as co-amorphous excipients for simvastatin and glibenclamide: physical properties and stability. Molecular Pharmaceutics, 2014, 11: 2381-2389.

4. Macfhionnghaile P, Hu Y, Gniado K, et al. Effects of ball-milling and cryomilling on sulfamerazine polymorphs: a quantitative study. Journal of Pharmaceutical Sciences, 2014, 103: 1766-1778.

5. Martínez L M, Videa M, López-Silva G A, et al. Stabilization of amorphous paracetamol based systems using traditional and novel strategies. International Journal of Pharmaceutics, 2014, 477: 294-305.

6. Pajula K, Wittoek L, Lehto V P, et al. Phase separation in coamorphous systems: in silico prediction and the experimental challenge of detection. Molecular Pharmaceutics, 2014, 11: 2271-2279.

7. Hu Y, Gniado K, Erxleben A, et al. Mechanochemical reaction of sulfathiazole with carboxylic acids: Formation of a cocrystal, a salt, and coamorphous solids. Crystal Growth & Design, 2014, 14: 803-813.

8. Suresh K, Mannava M K C, Nangia A. A novel curcumin–artemisinin coamorphous solid: physical properties and pharmacokinetic profile. Rsc Advances, 2014, 4: 58357-58361.

9. Bounartzi M, Panagopoulou A, Kantiranis N, et al. Effect of plasticiser type on the hot melt extrusion of venlafaxine hydrochloride. Journal of Pharmacy and Pharmacology, 2014, 66: 297-308.

Year 2015

1. Ali A M A, Ali A A, Maghrabi I A. Clozapine-carboxylic acid plasticized co-amorphous dispersions: Preparation, characterization and solution stability evaluation. Acta Pharmaceutica, 2015, 65: 133-146.

2. Beyer A, Grohganz H, Löbmann K, et al. Multivariate quantification of the solid state phase composition of co-amorphous naproxen-indomethacin. Molecules, 2015, 20: 19571-19587.

3. Craye G, Löbmann K, Grohganz H, et al. Characterization of amorphous and co-amorphous simvastatin formulations prepared by spray drying. Molecules, 2015, 20: 21532-21548.

4. Heikkinen A T, DeClerck L, Löbmann K, et al. Dissolution properties of co-amorphous drug-amino acid formulations in buffer and biorelevant media. Die Pharmazie-An International Journal of Pharmaceutical Sciences, 2015, 70: 452-457.

5. Jensen K T, Larsen F H, Cornett C, et al. Formation mechanism of coamorphous drug–amino acid mixtures. Molecular Pharmaceutics, 2015, 12: 2484-2492.

6. Knapik J, Wojnarowska Z, Grzybowska K, et al. Molecular dynamics and physical stability of coamorphous ezetimib and indapamide mixtures. Molecular Pharmaceutics, 2015, 12: 3610-3619.

7. Lenz E, Jensen K T, Blaabjerg L I, et al. Solid-state properties and dissolution behaviour of tablets containing co-amorphous indomethacin–arginine. European Journal of Pharmaceutics and Biopharmaceutics, 2015, 96: 44-52.

8. Wickström H, Palo M, Rijckaert K, et al. Improvement of dissolution rate of indomethacin by inkjet printing. European Journal of Pharmaceutical Sciences, 2015, 75: 91-100.

9. Dengale S J, Hussen S S, Krishna B S M, et al. Fabrication, solid state characterization and bioavailability assessment of stable binary amorphous phases of Ritonavir with Quercetin. European Journal of Pharmaceutics and Biopharmaceutics, 2015, 89: 329-338.

10. Qian S, Heng W, Wei Y, et al. Coamorphous lurasidone hydrochloride–saccharin with charge-assisted hydrogen bonding interaction shows improved physical stability and enhanced dissolution with pH-independent solubility behavior. Crystal Growth & Design, 2015, 15: 2920-2928.

11. Teja A, Musmade P B, Khade A B, et al. Simultaneous improvement of solubility and permeability by fabricating binary glassy materials of talinolol with naringin: Solid state characterization, in-vivo in-situ evaluation. European Journal of Pharmaceutical Sciences, 2015, 78: 234-244.

Year 2016

1. Ali A M A, Al-Remawi M M A. Freeze dried quetiapine-nicotinamide binary solid dispersions: A new strategy for improving physicochemical properties and ex vivo diffusion. Journal of Pharmaceutics, 2016, 2016:1-11.

2. Beyer A, Radi L, Grohganz H, et al. Preparation and recrystallization behavior of spray-dried co-amorphous naproxen–indomethacin. European Journal of Pharmaceutics and Biopharmaceutics, 2016, 104: 72-81.

3. Beyer A, Grohganz H, Löbmann K, et al. Influence of the cooling rate and the blend ratio on the physical stability of co-amorphous naproxen/indomethacin. European Journal of Pharmaceutics and Biopharmaceutics, 2016, 109: 140-148.

4. Corner P A, Harburn J J, Steed J W, et al. Stabilisation of an amorphous form of ROY through a predicted co-former interaction. Chemical Communications, 2016, 52: 6537-6540.

5. Gniado K, Löbmann K, Rades T, et al. The influence of co-formers on the dissolution rates of co-amorphous sulfamerazine/excipient systems. International Journal of Pharmaceutics, 2016, 504: 20-26.

6. Jensen K T, Blaabjerg L I, Lenz E, et al. Preparation and characterization of spray-dried co-amorphous drug–amino acid salts. Journal of Pharmacy and Pharmacology, 2016, 68: 615-624.

7. Jensen K T, Larsen F H, Löbmann K, et al. Influence of variation in molar ratio on co-amorphous drug-amino acid systems. European Journal of Pharmaceutics and Biopharmaceutics, 2016, 107: 32-39.

8. Kasten G, Grohganz H, Rades T, et al. Development of a screening method for co-amorphous formulations of drugs and amino acids. European Journal of Pharmaceutical Sciences, 2016, 95: 28-35.

9. Lim A W, Löbmann K, Grohganz H, et al. Investigation of physical properties and stability of indomethacin–cimetidine and naproxen–cimetidine co-amorphous systems prepared by quench cooling, coprecipitation and ball milling. Journal of Pharmacy and Pharmacology, 2016, 68: 36-45.

10. Maher E M, Ali A M A, Salem H F, et al. In vitro/in vivo evaluation of an optimized fast dissolving oral film containing olanzapine co-amorphous dispersion with selected carboxylic acids. Drug Delivery, 2016, 23: 3088-3100.

11. Martínez L M, Videa M, Sosa N G, et al. Long-term stability of new co-amorphous drug binary systems: study of glass transitions as a function of composition and shelf time. Molecules, 2016, 21: 1712-1725.

12. Riekes M K, Engelen A, Appeltans B, et al. New perspectives for fixed dose combinations of poorly water-soluble compounds: a case study with ezetimibe and lovastatin. Pharmaceutical Research, 2016, 33: 1259-1275.

13. Russo M G, Sancho M I, Silva L M A, et al. Looking for the interactions between omeprazole and amoxicillin in a disordered phase. an experimental and theoretical study. Spectrochimica Acta Part A: Molecular and Biomolecular Spectroscopy, 2016, 156: 70-77.

14. Sovago I, Wang W, Qiu D, et al. Properties of the sodium naproxen-lactose-tetrahydrate co-crystal upon processing and storage. Molecules, 2016, 21: 509-522.

15. Ueda H, Muranushi N, Sakuma S, et al. A strategy for co-former selection to design stable co-amorphous formations based on physicochemical properties of non-steroidal inflammatory drugs. Pharmaceutical Research, 2016, 33: 1018-1029.

16. Wairkar S, Gaud R. Co-amorphous combination of nateglinide-metformin hydrochloride for dissolution enhancement. AAPS PharmSciTech, 2016, 17: 673-681.

17. Yamamoto K, Kojima T, Karashima M, et al. Physicochemical evaluation and developability assessment of co-amorphouses of low soluble drugs and comparison to the co-crystals. Chemical and Pharmaceutical Bulletin, 2016, 64: 1739-1746.

18. Han Y, Pan Y, Lv J, et al. Powder grinding preparation of co-amorphous β-azelnidipine and maleic acid combination: molecular interactions and physicochemical properties. Powder Technology, 2016, 291: 110-120.

19. Qian S, Li Z, Heng W, et al. Charge-assisted intermolecular hydrogen bond formed in coamorphous system is important to relieve the pH-dependent solubility behavior of lurasidone hydrochloride. RSC Advances, 2016, 6: 106396-106412.

Year 2017

1. Arnfast L, Kamruzzaman M, Löbmann K, et al. Melt extrusion of high-dose co-amorphous drug-drug combinations. Pharmaceutical Research, 2017, 34: 2689-2697.

2. Beyer A, Grohganz H, Löbmann K, et al. Improvement of the physicochemical properties of Co-amorphous naproxen-indomethacin by naproxen-sodium. International Journal of Pharmaceutics, 2017, 526: 88-94.

3. Bi Y, Xiao D, Ren S, et al. The binary system of ibuprofen-nicotinamide under nanoscale confinement: from cocrystal to coamorphous state. Journal of Pharmaceutical Sciences, 2017, 106: 3150-3155.

4. Haneef J, Chadha R. Drug-drug multicomponent solid forms: cocrystal, coamorphous and eutectic of three poorly soluble antihypertensive drugs using mechanochemical approach. AAPS PharmSciTech, 2017, 18: 2279-2290.

5. Huang Y, Zhang Q, Wang J R, et al. Amino acids as co-amorphous excipients for tackling the poor aqueous solubility of valsartan. Pharmaceutical Development and Technology, 2017, 22: 69-76.

6. Kasten G, Nouri K, Grohganz H, et al. Performance comparison between crystalline and co-amorphous salts of indomethacin-lysine. International Journal of Pharmaceutics, 2017, 533: 138-144.

7. Lenz E, Löbmann K, Rades T, et al. Hot melt extrusion and spray drying of co-amorphous indomethacin-arginine with polymers. Journal of Pharmaceutical Sciences, 2017, 106: 302-312.

8. Moinuddin S M, Ruan S, Huang Y, et al. Facile formation of co-amorphous atenolol and hydrochlorothiazide mixtures via cryo-genic-milling: enhanced physical stability, dissolution and pharmacokinetic profile. International Journal of Pharmaceutics, 2017, 532: 393-400.

9. Ojarinta R, Heikkinen A T, Sievänen E, et al. Dissolution behavior of co-amorphous amino acid-indomethacin mixtures: The ability of amino acids to stabilize the supersaturated state of indomethacin. European Journal of Pharmaceutics and Biopharmaceutics, 2017, 112: 85-95.

10. Ojarinta R, Lerminiaux L, Laitinen R. Spray drying of poorly soluble drugs from aqueous arginine solution. International Journal of Pharmaceutics, 2017, 532: 289-298.

11. Pan Y, Pang W, Lv J, et al. Solid state characterization of azelnidipine–oxalic acid co-crystal and co-amorphous complexes: The effect of different azelnidipine polymorphs. Journal of Pharmaceutical and Biomedical Analysis, 2017, 138: 302-315.

12. Pang W, Lv J, Du S, et al. Preparation of curcumin–piperazine coamorphous phase and fluorescence spectroscopic and density functional theory simulation studies on the interaction with bovine serum albumin. Molecular Pharmaceutics, 2017, 14: 3013-3024.

13. Petry I, Löbmann K, Grohganz H, et al. Solid state properties and drug release behavior of co-amorphous indomethacin-arginine tablets coated with Kollicoat® Protect. European Journal of Pharmaceutics and Biopharmaceutics, 2017, 119: 150-160.

14. Renuka, Singh S K, Gulati M, et al. Stable amorphous binary systems of glipizide and atorvastatin powders with enhanced disso-lution profiles: formulation and characterization. Pharmaceutical Development and Technology, 2017, 22: 13-25.

15. Skieneh J M, Sathisaran I, Dalvi S V, et al. Co-amorphous form of curcumin–folic acid dihydrate with increased dissolution rate. Crystal Growth & Design, 2017, 17: 6273-6280.

16. Ueda H, Kadota K, Imono M, et al. Co-amorphous formation induced by combination of tranilast and diphenhydramine hydro-chloride. Journal of Pharmaceutical Sciences, 2017, 106: 123-128.

17. Walker G, Römann P, Poller B, et al. Probing pharmaceutical mixtures during milling: The potency of low-frequency Raman spectroscopy in identifying disorder. Molecular Pharmaceutics, 2017, 14: 4675-4684.

18. Wang J, Chang R, Zhao Y, et al. Coamorphous loratadine-citric acid system with enhanced physical stability and bioavailability. AAPS Pharmscitech, 2017, 18: 2541-2550.

19. Sterren V B, Aiassa V, Garnero C, et al. Preparation of chloramphenicol/amino acid combinations exhibiting enhanced dissolution rates and reduced drug-induced oxidative stress. AAPS PharmSciTech, 2017, 18: 2910-2918.

20. Liu X, Zhou L, Zhang F. Reactive melt extrusion to improve the dissolution performance and physical stability of naproxen amorphous solid dispersions. Molecular Pharmaceutics, 2017, 14: 658-673.

Year 2018

1. Abdelquader M M, Essa E A, El Maghraby G M. Inhibition of Co-crystallization of olmesartan medoxomil and hydrochlorothiazide for enhanced dissolution rate in their fixed dose combination. AAPS PharmSciTech, 2019, 20: 1-12.

2. Ainurofiq A, Mauludin R, Mudhakir D, et al. A novel desloratadine-benzoic acid co-amorphous solid: Preparation, characterization, and stability evaluation. Pharmaceutics, 2018, 10: 85.

3. An J H, Lim C, Kiyonga A N, et al. Co-amorphous screening for the solubility enhancement of poorly water-soluble mirabegron and investigation of their intermolecular interactions and dissolution behaviors. Pharmaceutics, 2018, 10: 149.

4. Anand V S K, Sakhare S D, Sree K S N, et al. The relevance of co-amorphous formulations to develop supersaturated dosage forms: In-vitro, and ex-vivo investigation of Ritonavir-Lopinavir co-amorphous materials. European Journal of Pharmaceutical Sciences, 2018, 123: 124-134.

5. Fung M H, DeVault M, Kuwata K T, et al. Drug-excipient interactions: effect on molecular mobility and physical stability of ketoconazole–organic acid coamorphous systems. Molecular Pharmaceutics, 2018, 15: 1052-1061.

6. Fung M, Bērziņš K, Suryanarayanan R. Physical stability and dissolution behavior of ketoconazole–organic acid coamorphous systems. Molecular Pharmaceutics, 2018, 15: 1862-1869.

7. Gniado K, MacFhionnghaile P, McArdle P, et al. The natural bile acid surfactant sodium taurocholate (NaTC) as a coformer in coamorphous systems: Enhanced physical stability and dissolution behavior of coamorphous drug-NaTc systems. International Journal of Pharmaceutics, 2018, 535: 132-139.

8. Kasten G, Lobo L, Dengale S, et al. In vitro and in vivo comparison between crystalline and co-amorphous salts of naproxen-arginine. European Journal of Pharmaceutics and Biopharmaceutics, 2018, 132: 192-199.

9. Kissi E O, Kasten G, Löbmann K, et al. The role of glass transition temperatures in coamorphous drug–amino acid formulations. Molecular Pharmaceutics, 2018, 15: 4247-4256.

10. Knapik-Kowalczuk J, Tu W, Chmiel K, et al. Co-Stabilization of amorphous pharmaceuticals—the case of nifedipine and nimodipine. Molecular Pharmaceutics, 2018, 15: 2455-2465.

11. Mannava M K, Suresh K, Kumar Bommaka M, et al. Curcumin-artemisinin coamorphous solid: Xenograft model preclinical study. Pharmaceutics, 2018, 10: 7-23.

12. Martínez-Jiménez C, Cruz-Angeles J, Videa M, et al. Co-amorphous simvastatin-nifedipine with enhanced solubility for possible use in combination therapy of hypertension and hypercholesterolemia. Molecules, 2018, 23(9): 2161-2174.

13. Meng-Lund H, Kasten G, Jensen K T, et al. The use of molecular descriptors in the development of co-amorphous formulations. European Journal of Pharmaceutical Sciences, 2018, 119: 31-38.

14. Mishra J, Rades T, Löbmann K, et al. Influence of solvent composition on the performance of spray-dried co-amorphous formulations. Pharmaceutics, 2018, 10(2): 47-60.

15. Mishra J, Löbmann K, Grohganz H, et al. Influence of preparation technique on co-amorphization of carvedilol with acidic amino acids. International Journal of Pharmaceutics, 2018, 552: 407-413.

16. Ojarinta R, Saarinen J, Strachan C J, et al. Preparation and characterization of multi-component tablets containing co-amorphous salts: Combining multimodal non-linear optical imaging with established analytical methods. European Journal of Pharmaceutics and Biopharmaceutics, 2018, 132: 112-126.

17. Petry I, Löbmann K, Grohganz H, et al. Undesired co-amorphisation of indomethacin and arginine during combined storage at high humidity conditions. International Journal of Pharmaceutics, 2018, 544: 172-180.

18. Petry I, Löbmann K, Grohganz H, et al. In situ co-amorphisation of arginine with indomethacin or furosemide during immersion in an acidic medium–A proof of concept study[J]. European Journal of Pharmaceutics and Biopharmaceutics, 2018, 133: 151-160.

19. Ruponen M, Visti M, Ojarinta R, et al. Permeability of glibenclamide through a PAMPA membrane: the effect of co-amorphization. European Journal of Pharmaceutics and Biopharmaceutics, 2018, 129: 247-256.

20. Russo M G, Baldoni H A, Dávila Y A, et al. Rational Design of a Famotidine–Ibuprofen Coamorphous System: An Experimental and Theoretical Study. The Journal of Physical Chemistry B, 2018, 122: 8772-8782.

21. Ueda H, Wu W, Löbmann K, et al. Application of a salt coformer in a co-amorphous drug system dramatically enhances the glass transition temperature: a case study of the ternary system carbamazepine, citric acid, and l-arginine. Molecular Pharmaceutics, 2018, 15: 2036-2044.

22. Wu W, Ueda H, Löbmann K, et al. Organic acids as co-formers for co-amorphous systems–Influence of variation in molar ratio on the physicochemical properties of the co-amorphous systems. European Journal of Pharmaceutics and Biopharmaceutics, 2018, 131: 25-32.

23. Wu W, Löbmann K, Schnitzkewitz J, et al. Aspartame as a co-former in co-amorphous systems. International Journal of Pharmaceutics, 2018, 549: 380-387.

24. Wu W, Löbmann K, Rades T, et al. On the role of salt formation and structural similarity of co-formers in co-amorphous drug delivery systems. International Journal of Pharmaceutics, 2018, 535: 86-94.

25. Zhu S, Gao H, Babu S, et al. Co-amorphous formation of high-dose zwitterionic compounds with amino acids to improve solubility and enable parenteral delivery. Molecular Pharmaceutics, 2018, 15: 97-107.

Year 2019

1. Hashim Ali K, Mohsin Ansari M, Ali Shah F, et al. Enhanced dissolution of valsartan-vanillin binary co-amorphous system loaded in mesoporous silica particles. Journal of Microencapsulation, 2019, 36: 10-20.

2. Aljohani M, MacFhionnghaile P, McArdle P, et al. Investigation of the formation of drug-drug cocrystals and coamorphous systems of the antidiabetic drug gliclazide. International Journal of Pharmaceutics, 2019, 561: 35-42.

3. Bohr A, Nascimento T L, Harmankaya N, et al. Efflux inhibitor bicalutamide increases oral bioavailability of the poorly soluble efflux substrate docetaxel in co-amorphous anti-cancer combination therapy. Molecules, 2019, 24: 266-279.

4. Chieng N, Teo X J, Cheah M H, et al. Molecular dynamics and physical stability of pharmaceutical co-amorphous systems: Correlation between structural relaxation times measured by Kohlrausch-Williams-Watts with the width of the glass transition temperature (ΔTg) and the onset of crystallization. Journal of Pharmaceutical Sciences, 2019, 108: 3848-3858.

5. Cruz-Angeles J, Videa M, Martínez L M. Highly soluble glimepiride and irbesartan co-amorphous formulation with potential application in combination therapy. AAPS PharmSciTech, 2019, 20: 1-12.

6. Elfakhri R, Burley J C. Investigation of potential amorphisation and co-amorphisation behaviour of the benzene di-carboxylic acids upon cryo-milling. Molecules, 2019, 24: 3990-4003.

7. Fung M H, Suryanarayanan R. Effect of organic acids on molecular mobility, physical stability, and dissolution of ternary ketoconazole spray-dried dispersions. Molecular Pharmaceutics, 2018, 16: 41-48.

8. Hirakawa Y, Ueda H, Miyano T, et al. New insight into transdermal drug delivery with supersaturated formulation based on co-amorphous system. International Journal of Pharmaceutics, 2019, 569: 118582-118589.

9. Kasten G, Löbmann K, Grohganz H, et al. Co-former selection for co-amorphous drug-amino acid formulations. International Journal of Pharmaceutics, 2019, 557: 366-373.

10. Kasten G, Duarte Í, Paisana M, et al. Process optimization and upscaling of spray-dried drug-amino acid co-amorphous formulations. Pharmaceutics, 2019, 11: 24-35.

11. Kissi E O, Khorami K, Rades T. Determination of Stable Co-Amorphous Drug–Drug Ratios from the Eutectic Behavior of Crystalline Physical Mixtures. Pharmaceutics, 2019, 11: 628-639.

12. Knapik-Kowalczuk J, Chmiel K, Jurkiewicz K, et al. Physical stability and viscoelastic properties of co-amorphous ezetimibe/simvastatin system. Pharmaceuticals, 2019, 12(1): 40-55.

13. Lababidi N, Kissi E O, Elgaher W A M, et al. Spray-drying of inhalable, multifunctional formulations for the treatment of biofilms formed in cystic fibrosis. Journal of Controlled Release, 2019, 314: 62-71.

14. Leng D, Kissi E O, Löbmann K, et al. Design of inhalable solid dosage forms of budesonide and theophylline for pulmonary combination therapy. AAPS Pharmscitech, 2019, 20: 1-11.

15. Liu W, Liu Y, Huang J, et al. Identification and investigation of the vibrational properties of crystalline and co-amorphous drugs with Raman and terahertz spectroscopy. Biomedical Optics Express, 2019, 10: 4290-4304.

16. Lodagekar A, Chavan R B, Mannava M K C, et al. Co amorphous valsartan nifedipine system: Preparation, characterization, in vitro and in vivo evaluation. European Journal of Pharmaceutical Sciences, 2019, 139: 105048-105057.

17. Lu W, Rades T, Rantanen J, et al. Inhalable co-amorphous budesonide-arginine dry powders prepared by spray drying. International Journal of Pharmaceutics, 2019, 565: 1-8.

18. Lu W, Rades T, Rantanen J, et al. Amino acids as stabilizers for spray-dried simvastatin powder for inhalation. International Journal of Pharmaceutics, 2019, 572: 118724-118736.

19. Mizoguchi R, Waraya H, Hirakura Y. Application of co-amorphous technology for improving the physicochemical properties of amorphous formulations. Molecular Pharmaceutics, 2019, 16: 2142-2152.

20. Pacult J, Rams-Baron M, Chmiel K, et al. How can we improve the physical stability of co-amorphous system containing flutamide and bicalutamide? The case of ternary amorphous solid dispersions. European Journal of Pharmaceutical Sciences, 2019, 136: 104947-104956.

21. Petry I, Löbmann K, Grohganz H, et al. In situ co-amorphisation in coated tablets–The combination of carvedilol with aspartic acid during immersion in an acidic medium. International Journal of Pharmaceutics, 2019, 558: 357-366.

22. Phan A D, Knapik-Kowalczuk J, Paluch M, et al. Theoretical model for the structural relaxation time in coamorphous drugs. Molecular Pharmaceutics, 2019, 16: 2992-2998.

23. Shi X, Song S, Ding Z, et al. Improving the solubility, dissolution, and bioavailability of Ibrutinib by preparing it in a coamorphous state with saccharin. Journal of Pharmaceutical Sciences, 2019, 108: 3020-3028.

24. Sormunen H, Ruponen M, Laitinen R. The effect of co-amorphization of glibenclamide on its dissolution properties and permeability through an MDCKII-MDR1 cell layer. International Journal of Pharmaceutics, 2019, 570: 118653-118664.

25. Wairkar S, Gaud R. Development and characterization of microstructured, spray-dried co-amorphous mixture of antidiabetic agents stabilized by silicate. AAPS PharmSciTech, 2019, 20: 1-10.

26. Wang R, Han J, Jiang A, et al. Involvement of metabolism-permeability in enhancing the oral bioavailability of curcumin in excipient-free solid dispersions co-formed with piperine. International Journal of Pharmaceutics, 2019, 561: 9-18.

27. Wang Z, Sun M, Liu T, et al. Co-amorphous solid dispersion systems of lacidipine-spironolactone with improved dissolution rate and enhanced physical stability. Asian Journal of Pharmaceutical Sciences, 2019, 14: 95-103.

28. Wei Y, Zhou S, Hao T, et al. Further enhanced dissolution and oral bioavailability of docetaxel by coamorphization with a natural P-gp inhibitor myricetin. European Journal of Pharmaceutical Sciences, 2019, 129: 21-30.

29. Wu W, Löbmann K, Schnitzkewitz J, et al. Dipeptides as co-formers in co-amorphous systems. European Journal of Pharmaceutics and Biopharmaceutics, 2019, 134: 68-76.

30. Wu W, Wang Y, Löbmann K, et al. Transformations between co-amorphous and co-crystal systems and their influence on the formation and physical stability of co-amorphous systems. Molecular Pharmaceutics, 2019, 16: 1294-1304.

Year 2020

1. Adhikari B R, Bērziņš K, Fraser-Miller S J, et al. Co-amorphization of kanamycin with amino acids improves aerosolization. Pharmaceutics, 2020, 12: 715-734.

2. da Costa N F, Fernandes A I, Pinto J F. Measurement of the amorphous fraction of olanzapine incorporated in a co-amorphous formulation. International Journal of Pharmaceutics, 2020, 588: 119716-119727.

3. Fael H, Demirel A L. Tannic acid as a co-former in co-amorphous systems: Enhancing their physical stability, solubility and dissolution behavior. International Journal of Pharmaceutics, 2020, 581: 119284-119294.

4. França M T, Marcos T M, Pereira R N, et al. Could the small molecules such as amino acids improve aqueous solubility and stabilize amorphous systems containing griseofulvin? European Journal of Pharmaceutical Sciences, 2020, 143: 105178-105786.

5. Heng W, Su M, Cheng H, et al. Incorporation of complexation into a coamorphous system dramatically enhances dissolution and eliminates gelation of amorphous lurasidone hydrochloride. Molecular Pharmaceutics, 2019, 17: 84-97.

6. Kilpeläinen T, Pajula K, Ervasti T, et al. Raman imaging of amorphous-amorphous phase separation in small molecule co-amorphous systems. European Journal of Pharmaceutics and Biopharmaceutics, 2020, 155: 49-54.

7. Liu J, Rades T, Grohganz H. Determination of the optimal molar ratio in amino acid-based co-amorphous systems. Molecular Pharmaceutics, 2020, 17: 1335-1342.

8. Liu J, Grohganz H, Rades T. Influence of polymer addition on the amorphization, dissolution and physical stability of co-amorphous systems. International Journal of Pharmaceutics, 2020, 588: 119768-119777.

9. Mohammed A, Zurek J, Madueke S, et al. Generation of high dose inhalable effervescent dispersions against pseudomonas aeruginosa biofilms. Pharmaceutical Research, 2020, 37: 1-14.

10. Moinuddin S M, Shi Q, Tao J, et al. Enhanced physical stability and synchronized release of febuxostat and indomethacin in co-amorphous solids. AAPS PharmSciTech, 2020, 21: 1-10.

11. Pajula K, Hyyryläinen J, Koistinen A, et al. Detection of amorphous-amorphous phase separation in small molecular co-amorphous mixtures with SEM-EDS. European Journal of Pharmaceutics and Biopharmaceutics, 2020, 150: 43-49.

12. Park H, Seo H J, Hong S, et al. Characterization and therapeutic efficacy evaluation of glimepiride and L-arginine co-amorphous formulation prepared by supercritical antisolvent process: Influence of molar ratio and preparation methods. International Journal of Pharmaceutics, 2020, 581: 119232-119245.

13. Ruponen M, Rusanen H, Laitinen R. Dissolution and permeability properties of co-amorphous formulations of hydrochlorothiazide. Journal of Pharmaceutical Sciences, 2020, 109: 2252-2261.

14. Tawfeek H M, Chavan T, Kunda N K. Effect of spray drying on amorphization of indomethacin nicotinamide cocrystals: optimization, characterization, and stability study. AAPS PharmSciTech, 2020, 21: 1-13.

15. Ueda H, Bøtker J P, Edinger M, et al. Formulation of co-amorphous systems from naproxen and naproxen sodium and in situ monitoring of physicochemical state changes during dissolution testing by Raman spectroscopy. International Journal of Pharmaceutics, 2020, 587: 119662-119672.

16. Wang M, Liu S, Jia L, et al. Exploring the physical stability of three nimesulide–indomethacin co-amorphous systems from the perspective of molecular aggregates. European Journal of Pharmaceutical Sciences, 2020, 147: 105294-105304.

17. Wang S, Heng W, Wang X, et al. Coamorphization combined with complexation enhances dissolution of lurasidone hydrochloride and puerarin with synchronized release. International Journal of Pharmaceutics, 2020, 588: 119793-119806.

18. Wostry M, Plappert H, Grohganz H. Preparation of co-amorphous systems by freeze-drying. Pharmaceutics, 2020, 12: 941-953.

19. Yu D, Kan Z, Shan F, et al. Triple strategies to improve oral bioavailability by fabricating coamorphous forms of ursolic acid with piperine: enhancing water-solubility, permeability, and inhibiting cytochrome P450 isozymes. Molecular Pharmaceutics, 2020, 17: 4443-4462.

20. Zhang M, Suo Z, Peng X, et al. Microcrystalline cellulose as an effective crystal growth inhibitor for the ternary Ibrutinib formulation. Carbohydrate Polymers, 2020, 229: 115476-115485.

21. Zhang Y, Gao Y, Du X, et al. Combining co-amorphous-based spray drying with inert carriers to achieve improved bioavailability and excellent downstream manufacturability. Pharmaceutics, 2020, 12: 1063-1080.

22. Nair A, Varma R, Gourishetti K, et al. Influence of preparation methods on physicochemical and pharmacokinetic properties of co-amorphous formulations: the case of co-amorphous atorvastatin: naringin. Journal of Pharmaceutical Innovation, 2020, 15: 365-379.

Year 2021

1. Chambers L I, Grohganz H, Palmelund H, et al. Predictive identification of co-formers in co-amorphous systems. European Journal of Pharmaceutical Sciences, 2021, 157: 105636-105645.

2. Veith H, Wiechert F, Luebbert C, et al. Combining crystalline and polymeric excipients in API solid dispersions–Opportunity or risk?. European Journal of Pharmaceutics and Biopharmaceutics, 2021, 158: 323-335.

3. Wu W, Grohganz H, Rades T, et al. Comparison of co-former performance in co-amorphous formulations: Single amino acids, amino acid physical mixtures, amino acid salts and dipeptides as co-formers. European Journal of Pharmaceutical Sciences, 2021, 156: 105582-105590.

4. Hirakawa Y, Ueda H, Takata Y, et al. Co-amorphous formation of piroxicam-citric acid to generate supersaturation and improve skin permeation. European Journal of Pharmaceutical Sciences, 2021, 158: 105667-105673.

5. Otsuka Y, Kuwashima W, Tanaka Y, et al. Effects of heat treatment on indomethacin-cimetidine mixture; Investigation of drug-drug interaction using singular value decomposition in FTIR spectroscopy. Journal of Pharmaceutical Sciences, 2021, 110: 1142-1147.
