# Supplementary material for: Co-Amorphous Drug Formulations in Numbers: Recent Advances in Co-Amorphous Drug Formulations with Focus on Co-Formability, Molar Ratio, Preparation Methods, Physical Stability, In Vitro and In Vivo Performance, and New Formulation Strategies
Source: Pharmaceutics. 2021 Mar 15;13(3):389. doi: 10.3390/pharmaceutics13030389 (PMC7999207; doi:10.3390/pharmaceutics13030389)
Supplement: Supplementary file 1 [file pharmaceutics-13-00389-s001.zip › Appendix C.pdf]

Appendix C. List of review articles focusing on co-amorphous drug formulations

Title	Authors	Year	Publication
Co amorphous systems: A product development perspective	Rahul B. Chavan, Rajesh Thipparaboina, Dinesh Kumar, Nalini R. Shastri	2016	International Journal of Pharmaceutics, 515, 403-415.
Recent advances in co-amorphous drug formulations	Swapnil Jayant Dengale, Holger Grohganz, Thomas Rades, Korbinian Löbmann	2016	Advanced Drug Delivery Reviews, 100, 116-125.
Rational excipient selection for co-amorphous formulations	Ossi Korhonen, Katja Pajula, Riikka Laitinen	2017	Expert Opinion on Drug Delivery, 14, 551-569.
Supersaturating drug delivery systems: The potential of co-amorphous drug formulations	Riikka Laitinen, Korbinian Löbmann, Holger Grohganz, Petra Priemel, Clare J. Strachan, Thomas Rades	2017	International Journal of Pharmaceutics, 532, 1-12.
Co-amorphous solid dispersions for solubility and absorption improvement of drugs: Composition, preparation, characterization and formulations for oral delivery	Anna Karagianni, Kyriakos Kachrimanis, Ioannis Nikolakakis	2018	Pharmaceutics, 10, 98-123.
Co-amorphous active pharmaceutical ingredient-small molecule mixtures: Considerations in the choice of co-formers for enhancing dissolution and oral bioavailability	Ann Newman, Susan M. Reutzel-Edens, George Zograf	2018	Journal of Pharmaceutical Sciences, 107, 5-17.
Advances in co-amorphous drug delivery systems	Qin Shi, Sakib M. Moinuddin, Ting Cai	2019	Acta Pharmaceutica Sinica B, 9, 19-35.
Co-amorphous systems for the delivery of poorly water-soluble drugs: recent advances and an update	Jiawei Han, Yuanfeng Wei, Yan Lu, Runze Wang, Jianjun Zhang, Yuan Gao, Shuai Qian	2020	Expert Opinion on Drug Delivery, 17, 1411-1435.
