# Supplementary material for: Co-Amorphous Drug Formulations in Numbers: Recent Advances in Co-Amorphous Drug Formulations with Focus on Co-Formability, Molar Ratio, Preparation Methods, Physical Stability, In Vitro and In Vivo Performance, and New Formulation Strategies
Source: Pharmaceutics. 2021 Mar 15;13(3):389. doi: 10.3390/pharmaceutics13030389 (PMC7999207; doi:10.3390/pharmaceutics13030389)
Supplement: Supplementary file 1 [file pharmaceutics-13-00389-s001.zip › Appendix D.pdf]

Appendix D. Overview of studies discussing phospholipids and sugars as excipients for CAMS

Drug	Co-former	Co-former type	Preparation	Amorphous	References
Ibuprofen	Phospholipon 100	Phospholipids	Solvent evaporation	N	[1]
Ibuprofen	Phospholipon 100H	Phospholipids	Solvent evaporation	N	[1]
Ibuprofen	Dimyristoylphosphatidylglycerol (DMPG)	Phospholipids	Solvent evaporation	N	[1]
Ibuprofen	Dimyristoylphosphatidylcholine (DMPC)	Phospholipids	Solvent evaporation	N	[1]
Ibuprofen	Dipalmitoylphosphatidylcholine (DPPC)	Phospholipids	Solvent evaporation	N	[1]
Ibuprofen	Distearoylphosphatidylcholine (DSPC)	Phospholipids	Solvent evaporation	N	[1]
Ibuprofen	Phospholipon 100	Phospholipids	Solvent evaporation	N	[1]
Albendazole	Monoacyl PC	Phospholipids	Solvent evaporation	N	[2]
Celecoxib	Monoacyl PC	Phospholipids	Solvent evaporation	Y	[2]
Ezetimibe	Monoacyl PC	Phospholipids	Solvent evaporation	Y	[2]
Furosemide	Monoacyl PC	Phospholipids	Solvent evaporation	Y	[2]
Glibenclamide	Monoacyl PC	Phospholipids	Solvent evaporation	N	[2]
Indomethacin	Monoacyl PC	Phospholipids	Solvent evaporation	Y	[2]

Ketoprofen	Monoacyl PC	Phospholip-ids	Solvent evaporation	Y	[2]
Mebendazole	Monoacyl PC	Phospholip-ids	Solvent evaporation	N	[2]
Mefenamic acid	Monoacyl PC	Phospholip-ids	Solvent evaporation	N	[2]
Naproxen	Monoacyl PC	Phospholip-ids	Solvent evaporation	N	[2]
Phenytoin	Monoacyl PC	Phospholip-ids	Solvent evaporation	N	[2]
Probucol	Monoacyl PC	Phospholip-ids	Solvent evaporation	N	[2]
Sulfasalazine	Monoacyl PC	Phospholip-ids	Solvent evaporation	N	[2]
Sulfathiazole	Monoacyl PC	Phospholip-ids	Solvent evaporation	Y	[2]
Albendazole	Diacyl PC	Phospholip-ids	Solvent evaporation	N	[2]
Celecoxib	Diacyl PC	Phospholip-ids	Solvent evaporation	Y	[2]
Ezetimibe	Diacyl PC	Phospholip-ids	Solvent evaporation	Y	[2]
Furosemide	Diacyl PC	Phospholip-ids	Solvent evaporation	Y	[2]
Glibenclamide	Diacyl PC	Phospholip-ids	Solvent evaporation	N	[2]
Indomethacin	Diacyl PC	Phospholip-ids	Solvent evaporation	Y	[2]
Ketoprofen	Diacyl PC	Phospholip-ids	Solvent evaporation	Y	[2]
Mebendazole	Diacyl PC	Phospholip-ids	Solvent evaporation	N	[2]
Mefenamic acid	Diacyl PC	Phospholip-ids	Solvent evaporation	N	[2]

Naproxen	Diacyl PC	Phospholip-ids	Solvent evaporation	Y	[2]
Phenytoin	Diacyl PC	Phospholip-ids	Solvent evaporation	N	[2]
Probucol	Diacyl PC	Phospholip-ids	Solvent evaporation	N	[2]
Sulfasalazine	Diacyl PC	Phospholip-ids	Solvent evaporation	N	[2]
Sulfathiazole	Diacyl PC	Phospholip-ids	Solvent evaporation	Y	[2]
Curcumin	Chitosan oligosaccharide	Sugars	Ball milling	Y	[3]
Indomethacin	Octaacetylmaltose	Sugars	Melt-quench	Y	[4]
Indomethacin	Octaacetylsucrose	Sugars	Melt-quench	Y	[4]
Indomethacin	Pentaacetylglucose	Sugars	Melt-quench	Y	[4]
Indomethacin	Octaacetylmaltose	Sugars	Melt-quench	Y	[5]
Naproxen	Acetylated maltose	Sugars	Melt-quench	Y	[6]
Naproxen	Acetylated sucrose	Sugars	Melt-quench	Y	[6]
Itraconazole	Octaacetylmaltose	Sugars	Melt-quench	Y	[7]
Itraconazole	Octaacetylsucrose	Sugars	Melt-quench	Partly amor-phous	[7]
Itraconazole	Pentaacetylglucose	Sugars	Melt-quench	Partly amor-phous	[7]
Nifedipine	Octaacetylmaltose	Sugars	Melt-quench	Y	[8]
Nifedipine	Octaacetylsucrose	Sugars	Melt-quench	Y	[8]
Nifedipine	Pentaacetylglucose	Sugars	Melt-quench	N	[8]
Nifedipine	Pentaacetylgalactose	Sugars	Melt-quench	N	[8]
Clotrimazole	Mannitol	Sugars	Fusion process	N	[9]
Clotrimazole	Fructose	Sugars	Fusion process	N	[9]
Clotrimazole	Dextrose	Sugars	Fusion process	Not mentioned	[9]
Clotrimazole	Maltose	Sugars	Fusion process	Not mentioned	[9]
Indomethacin	Maltose	Sugars	Vacuum drying	Y	[10]
Indomethacin	Trehalose	Sugars	Vacuum drying	Y	[10]
Indomethacin	Maltitol	Sugars	Vacuum drying	Y	[10]

Cinnamalde-hyde	Maltose	Sugars	Vacuum drying	Y	[11]
Indomethacin	Glucose	Sugars	Melt-quench	Y	[12]
Carbamazepine	D-glucosamide hydrochloride	Sugars	Solvent evaporation	N	[13]
Nifedipine	Mannitol	Sugars	Melt-quench	Y	[14]
Etoricoxib	Lactose	Sugars	Solvent evaporation	N	[15]
Etoricoxib	Sucrose	Sugars	Solvent evaporation	N	[15]
Etoricoxib	Mannitol	Sugars	Solvent evaporation	N	[15]
Sodium naproxen	Lactose	Sugars	Spray drying and ball milling	Y	[16]
Curcumin	Dextrose	Sugars	Lag	N	[17]
Sulfamerazine	Trehalose	Sugars	Cyromilling	N	[18]
Nifedipine	Sucrose	Sugars	Melt-quench	N	[19]
Probucol	Sucrose	Sugars	Melt-quench	N	[19]
Itraconazole	Sucrose	Sugars	Melt-quench	N	[19]
Indomethacin	Sucrose	Sugars	Melt-quench	N	[19]
Clotrimazole	Sucrose	Sugars	Melt-quench	N	[19]
Carvedilol	Sucrose	Sugars	Melt-quench	N	[19]
Celecoxib	Octaacetylmaltose	Sugars	Melt-quench	Y	[20]
Sucrose	Dextran	Sugars	Freeze drying	Y	[21]
Sucrose	Trehalose	Sugars	Freeze drying	Y	[21]

Reference

1. Hussain, M.D.; Saxena, V.; Brausch, J.F.; Talukder, R.M. Ibuprofen-phospholipid solid dispersions: improved dissolution and gastric tolerance. *Int J Pharm* **2012**, *422*, 290-294.
2. Gautschi, N.; Van Hoogevest, P.; Kuentz, M. Amorphous drug dispersions with mono- and diacyl lecithin: On molecular categorization of their feasibility and UV dissolution imaging. *Int J Pharm* **2015**, *491*, 218-230.
3. Huang, R.; Han, J.; Wang, R.; Zhao, X.; Qiao, H.; Chen, L.; Li, W.; Di, L.; Zhang, W.; Li, J. Surfactant-free solid dispersion of BCS class IV drug in an amorphous chitosan oligosaccharide matrix for concomitant dissolution in vitro - permeability increase. *Eur J Pharm Sci* **2019**, *130*, 147-155.
4. Kaminska, E.; Adrjanowicz, K.; Tarnacka, M.; Kolodziejczyk, K.; Dulski, M.; Mapesa, E.U.; Zakowiecki, D.; Hawelek, L.; Kaczmarczyk-Sedlak, I.; Kaminski, K. Impact of inter- and intra-molecular interactions on the physical stability of indomethacin dispersed in acetylated saccharides. *Mol Pharm* **2014**, *11*, 2935-2947.
5. Kaminska, E.; Adrjanowicz, K.; Zakowiecki, D.; Milanowski, B.; Tarnacka, M.; Hawelek, L.; Dulski, M.; Pilch, J.; Smolka, W.; Kaczmarczyk-Sedlak, I., et al. Enhancement of the physical stability of amorphous indomethacin by mixing it with octaacetylmaltose. inter and intra molecular studies. *Pharm Res* **2014**, *31*, 2887-2903.
6. Kaminska, E.; Madejczyk, O.; Tarnacka, M.; Jurkiewicz, K.; Kaminski, K.; Paluch, M. Studying of crystal growth and overall crystallization of naproxen from binary mixtures. *Eur J Pharm Biopharm* **2017**, *113*, 75-87.

7. Kaminska, E.; Tarnacka, M.; Kolodziejczyk, K.; Dulski, M.; Zakowiecki, D.; Hawelek, L.; Adrjanowicz, K.; Zych, M.; Garbacz, G.; Kaminski, K. Impact of low molecular weight excipient octaacetylmaltose on the liquid crystalline ordering and molecular dynamics in the supercooled liquid and glassy state of itraconazole. *Eur J Pharm Biopharm* **2014**, *88*, 1094-1104.
8. Kaminska, E.; Tarnacka, M.; Wlodarczyk, P.; Jurkiewicz, K.; Kolodziejczyk, K.; Dulski, M.; Haznar-Garbacz, D.; Hawelek, L.; Kaminski, K.; Wlodarczyk, A., et al. Studying the impact of modified saccharides on the molecular dynamics and crystallization tendencies of model api nifedipine. *Mol Pharm* **2015**, *12*, 3007-3019.
9. Madgulkar, A.; Bandivadekar, M.; Shid, T.; Rao, S. Sugars as solid dispersion carrier to improve solubility and dissolution of the BCS class II drug: clotrimazole. *Drug Dev Ind Pharm* **2015**, *42*, 28-38.
10. Takeda, K.; Gotoda, Y.; Hirota, D.; Hidaka, F.; Sato, T.; Matsuura, T.; Imanaka, H.; Ishida, N.; Imamura, K. Surfactant-free solid dispersions of hydrophobic drugs in an amorphous sugar matrix dried from an organic solvent. *Mol Pharm* **2017**, *14*, 791-798.
11. Satoh, T.; Hidaka, F.; Miyake, K.; Yoshiyama, N.; Takeda, K.; Matsuura, T.; Imanaka, H.; Ishida, N.; Imamura, K. Surfactant-free solid dispersion of fat-soluble flavour in an amorphous sugar matrix. *Food Chem* **2016**, *197*, 1136-1142.
12. Martinez, L.M.; Videa, M.; Lopez Silva, T.; Castro, S.; Caballero, A.; Lara-Diaz, V.J.; Castorena-Torres, F. Two-phase amorphous-amorphous solid drug dispersion with enhanced stability, solubility and bioavailability resulting from ultrasonic dispersion of an immiscible system. *Eur J Pharm Biopharm* **2017**, *119*, 243-252.
13. Al-Hamidi, H.; Edwards, A.A.; Mohammad, M.A.; Nokhodchi, A. To enhance dissolution rate of poorly water-soluble drugs: glucosamine hydrochloride as a potential carrier in solid dispersion formulations. *Colloids Surf B Biointerfaces* **2010**, *76*, 170-178.
14. Zajc, N.; Obreza, A.; Bele, M.; Srcic, S. Physical properties and dissolution behaviour of nifedipine/mannitol solid dispersions prepared by hot melt method. *Int J Pharm* **2005**, *291*, 51-58.
15. Das, A.; Nayak, A.K.; Mohanty, B.; Panda, S. Solubility and dissolution enhancement of etoricoxib by solid dispersion technique using sugar carriers. *ISRN Pharm* **2011**, *1*, 819765.
16. Sovago, I.; Wang, W.; Qiu, D.; Raijada, D.; Rantanen, J.; Grohganz, H.; Rades, T.; Bond, A.D.; Lobmann, K. Properties of the sodium naproxen-lactose-tetrahydrate co-crystal upon processing and storage. *Molecules* **2016**, *21*, 509.
17. Skieneh, J.M.; Sathisaran, I.; Dalvi, S.V.; Rohani, S. Co-amorphous form of curcumin–folic acid dihydrate with increased dissolution rate. *Cryst Growth Des* **2017**, *17*, 6273-6280.
18. Macfhionnghaile, P.; Hu, Y.; Gniado, K.; Curran, S.; McArdle, P.; Erxleben, A. Effects of ball-milling and cryomilling on sulfamerazine polymorphs: a quantitative study. *J Pharm Sci* **2014**, *103*, 1766-1778.
19. Mizoguchi, R.; Waraya, H.; Hirakura, Y. Application of co-amorphous technology for improving the physicochemical properties of amorphous formulations. *Mol Pharm* **2019**, *16*, 2142-2152.
20. Grzybowska, K.; Paluch, M.; Wlodarczyk, P.; Grzybowski, A.; Kaminski, K.; Hawelek, L.; Zakowiecki, D.; Kasprzycka, A.; Jankowska-Sumara, I. Enhancement of amorphous celecoxib stability by mixing it with octaacetylmaltose: the molecular dynamics study. *Mol Pharm* **2012**, *9*, 894-904.
21. Zografi, S.L.S.G. Enthalpy relaxation in binary amorphous mixtures containing sucrose. *Pharm Res* **1998**, *15*, 1828-1834.
