# Supplementary material for: Co-Amorphous Drug Formulations in Numbers: Recent Advances in Co-Amorphous Drug Formulations with Focus on Co-Formability, Molar Ratio, Preparation Methods, Physical Stability, In Vitro and In Vivo Performance, and New Formulation Strategies
Source: Pharmaceutics. 2021 Mar 15;13(3):389. doi: 10.3390/pharmaceutics13030389 (PMC7999207; doi:10.3390/pharmaceutics13030389)
Supplement: Supplementary file 1 [file pharmaceutics-13-00389-s001.zip › Supplementary Materials.pdf]

pharmaceutics

Supplementary Materials: Co-Amorphous Drug Formulations in Numbers: Recent Advances in Co-Amorphous Drug Formulations with Focus on Co-Formability, Molar Ratio, Preparation Methods, Physical Stability, In Vitro and In Vivo Performance, and New Formulation Strategies

Jingwen Liu, Holger Grohganz, Korbinian Löbmann, Thomas Rades and Nele-Johanna Hempel

Table S1. Overview of screened CAMS and non-CAMS with amino acids as co-formers. (y: yes. n: no. depends: different outcome depending on the used preparation method and/or parameters of the preparation method).

Drug	Co-former	CAMS (y/no/depends)	References
**Budesonide**	Arginine	y	[1]
**Carbamazepine**	Arginine	n (ball milling)	[2–6]
**Carbamazepine**	Arginine-Tyrosin	n (ball milling)	[2]
**Carbamazepine**	Phenylalanine-Tryptophan	y	[2]
**Carbamazepine**	Arginine-Tryptophan	y	[2]
**Carbamazepine**	Tryptophan-Tyrosine	n (ball milling)	[2]
**Carbamazepine**	Aspartic acid	n (ball milling)	[3–5]
**Carbamazepine**	Cysteine	n (ball milling)	[3–5]
**Carbamazepine**	Glutamic acid	n (ball milling)	[3–5]
**Carbamazepine**	Glycine	n (ball milling)	[3–5]
**Carbamazepine**	Histidine	n (ball milling)	[3–5]
**Carbamazepine**	Lysine	n (ball milling)	[3–5]
**Carbamazepine**	Proline	n (ball milling)	[3–5]
**Carbamazepine**	Phenylalanine	n (ball milling)	[2–5]
**Carbamazepine**	Tryptophan	y	[2–5,7]
**Carbamazepine**	Alanine	n (ball milling)	[3–5]
**Carbamazepine**	Asparagine	n (ball milling)	[3–5]
**Carbamazepine**	Glutamine	n (ball milling)	[3–5]
**Carbamazepine**	Isoleucine	n (ball milling)	[3–5]
**Carbamazepine**	Leucine	n (ball milling)	[3–5]
**Carbamazepine**	Methionine	n (ball milling)	[3–5]
**Carbamazepine**	Serine	n (ball milling)	[3–5]
**Carbamazepine**	Threonine	n (ball milling)	[3–5]
**Carbamazepine**	Tyrosine	n (ball milling)	[2–5]
**Carbamazepine**	Valine	n (ball milling)	[3–5]
**Carvedilol**	Arginine	n (ball milling)	[3–5]
**Carvedilol**	Aspartic acid	depends	[3–5,8–11]
**Carvedilol**	Cysteine	n (ball milling)	[3–5]
**Carvedilol**	Glutamic acid	depends	[3–5,8]
**Carvedilol**	Glycine	n (ball milling)	[3–5]
**Carvedilol**	Histidine	n (ball milling)	[3–5]
**Carvedilol**	Lysine	n (ball milling)	[3–5]
**Carvedilol**	Proline	n (ball milling)	[3–5]
**Carvedilol**	Phenylalanine	y	[3–5,7]
**Carvedilol**	Tryptophan	y	[3–5,7,12]
**Carvedilol**	Alanine	n (ball milling)	[3–5]
**Carvedilol**	Asparagine	n (ball milling)	[3–5]
**Carvedilol**	Glutamine	n (ball milling)	[3–5]

**Carvedilol**	Isoleucine	y	[3–5,7]
**Carvedilol**	Leucine	y	[3–5,7]
**Carvedilol**	Methionine	y	[3–5,7]
**Carvedilol**	Serine	n (ball milling)	[3–5]
**Carvedilol**	Threonine	n (ball milling)	[3–5]
**Carvedilol**	Tyrosine	n (ball milling)	[3–5]
**Carvedilol**	Valine	y	[3–5,7]
**Chloramphenicol**	Arginine	n (freeze drying)	[13]
**Chloramphenicol**	Cysteine	n (freeze drying)	[13]
**Chloramphenicol**	Glycine	n (freeze drying)	[13]
**Chloramphenicol**	Leucine	n (freeze drying)	[13]
**Cimetidine**	Arginine	y	[14]
**Cimetidine**	Citrulline	n (ball milling)	[14]
**Furosemide**	Arginine	y	[3–5,7,14–16]
**Furosemide**	Aspartic acid	n (ball milling)	[3–5]
**Furosemide**	Cysteine	y	[3–5,7]
**Furosemide**	Glutamic acid	n (ball milling)	[3–5]
**Furosemide**	Glycine	n (ball milling)	[3–5]
**Furosemide**	Histidine	y	[3–5,7]
**Furosemide**	Lysine	y	[3–5,7]
**Furosemide**	Proline	y	[3–5,7]
**Furosemide**	Phenylalanine	y	[3–5,7]
**Furosemide**	Tryptophan	y	[3–5,7,16,17]
**Furosemide**	Alanine	n (ball milling)	[3–5]
**Furosemide**	Asparagine	n (ball milling)	[3–5]
**Furosemide**	Glutamine	n (ball milling)	[3–5]
**Furosemide**	Isoleucine	y	[3–5,7]
**Furosemide**	Leucine	y	[3–5,7]
**Furosemide**	Methionine	y	[3–5,7]
**Furosemide**	Serine	n (ball milling)	[3–5]
**Furosemide**	Threonine	n (ball milling)	[3–5]
**Furosemide**	Tyrosine	n (ball milling)	[3–5]
**Furosemide**	Valine	y	[3–5,7]
**Furosemide**	Citrulline	y	[14]
**Glibenclamide**	Aspartic acid	n (ball milling)	[18]
**Glibenclamide**	Lysine	n (ball milling)	[18]
**Glibenclamide**	Serine	y	[18–20]
**Glibenclamide**	Threonine	y	[18,19]
**Glibenclamide**	Serin-Threonine	y	[18,19]
**Glibenclamide**	Arginine	y	[20,21]
**Glibenclamide**	Arginine-Serine	y	[21]
**Glibenclamide**	Arginine-Sodium lauryl sulfate	y	[20]
**Glimepiride**	Arginine	depends	[22]
**Griseofulvin**	Aspartic acid	n (ball milling)	[23]
**Griseofulvin**	Valine	n (ball milling)	[23]
**Griseofulvin**	Methionine	n (ball milling)	[23]
**Griseofulvin**	Lysine	n (ball milling)	[23]
**Griseofulvin**	Tryptophan	y	[23]
**Hydrochlorothiazide**	Arginine	y	[24]
**Ibrutinib**	Glutamic acid	n (ball milling)	[25]
**Ibrutinib**	Arginine	n (ball milling)	[25]

**Ibrutinib**	Histidine	n (ball milling)	[25]
**Ibrutinib**	Glutamine	n (ball milling)	[25]
**Ibrutinib**	Valine	n (ball milling)	[25]
**Ibrutinib**	Tyrosine	n (ball milling)	[25]
**Ibrutinib**	Alanine	n (ball milling)	[25]
**Ibuprofen**	Arginine	y	[26,27]
**Indomethacin**	Arginine-Tyrosin	n (ball milling)	[2]
**Indomethacin**	Phenylalanine-Tryptophan	y	[2]
**Indomethacin**	Arginine-Phenylalanine	y	[2]
**Indomethacin**	Tryptophan-Tyrosine	n (ball milling)	[2]
**Indomethacin**	Arginine	y	[2–5,7,16,26–35]
**Indomethacin**	Aspartic acid	n (ball milling)	[3–5]
**Indomethacin**	Cysteine	n (ball milling)	[3–5]
**Indomethacin**	Glutamic acid	n (ball milling)	[3–5]
**Indomethacin**	Glycine	n (ball milling)	[3–5]
**Indomethacin**	Histidine	depends	[3–5,31,35]
**Indomethacin**	Lysine	depends	[3–5,7,31,35,36]
**Indomethacin**	Proline	y	[3–5,7]
**Indomethacin**	Phenylalanine	y	[2–5,7,29]
**Indomethacin**	Tryptophan	y	[2–5,7,12,16,17,29,37]
**Indomethacin**	Alanine	n (ball milling)	[3–5]
**Indomethacin**	Asparagine	n (ball milling)	[3–5]
**Indomethacin**	Glutamine	n (ball milling)	[3–5]
**Indomethacin**	Isoleucine	y	[3–5,7]
**Indomethacin**	Leucine	y	[3–5,7]
**Indomethacin**	Methionine	y	[3–5,7]
**Indomethacin**	Serine	n (ball milling)	[3–5]
**Indomethacin**	Threonine	n (ball milling)	[3–5]
**Indomethacin**	Tyrosine	n (ball milling)	[2–5]
**Indomethacin**	Valine	y	[3–5,7]
**Kanamycin sulfate**	Valine	y	[38]
**Kanamycin sulfate**	Methionine	y	[38]
**Kanamycin sulfate**	Phenylalanine	y	[38]
**Kanamycin sulfate**	Tryptophan	y	[38]
**Lurasidone hydrochloride**	Cysteine hydrochloride	y	[39]
**Mebendazole**	Arginine	n (ball milling)	[3–5,14,40]
**Mebendazole**	Aspartic acid	n (ball milling)	[3–5,41,42]
**Mebendazole**	Cysteine	n (ball milling)	[3–5]
**Mebendazole**	Glutamic acid	n (ball milling)	[3–5,40]
**Mebendazole**	Glycine	n (ball milling)	[3–5,42]
**Mebendazole**	Histidine	n (ball milling)	[3–5,42]
**Mebendazole**	Lysine	y	[3–5,7]
**Mebendazole**	Proline	depends	[3–5,42]
**Mebendazole**	Phenylalanine	depends	[3–5,7,41,42]
**Mebendazole**	Tryptophan	y	[3–5,7,42]
**Mebendazole**	Alanine	n (ball milling)	[3–5]
**Mebendazole**	Asparagine	n (ball milling)	[3–5]
**Mebendazole**	Glutamine	n (ball milling)	[3–5]
**Mebendazole**	Isoleucine	y	[3–5,7]
**Mebendazole**	Leucine	y	[3–5,7]
**Mebendazole**	Methionine	y	[3–5,7]

**Mebendazole**	Serine	n (ball milling)	[3–5]
**Mebendazole**	Threonine	n (ball milling)	[3–5]
**Mebendazole**	Tyrosine	n (ball milling)	[3–5,42]
**Mebendazole**	Valine	n (ball milling)	[3–5]
**Mebendazole**	Aspartic acid-Phenylalanine	n (ball milling)	[41]
**Mebendazole**	Citrulline	n (ball milling)	[14]
**Mebendazole**	Tryptophan-Phenylalanine	y	[42]
**Mebendazole**	Aspartic acid-Tyrosine	n (ball milling)	[42]
**Mebendazole**	Histidine-Glycine	n (ball milling)	[42]
**Mebendazole**	Proline-Tryptophan	y	[42]
**Naproxen**	Arginine	y	[43–45]
**Naproxen**	Tryptophan	n (ball milling)	[43]
**Naproxen**	Arginine-Prolin	y	[43]
**Naproxen**	Tryptophan-Prolin	y	[43]
**Naproxen**	Lysine	y	[44]
**Naproxen**	Arginine-Sodium dodecyl sulfate	n (freeze drying)	[44]
**Naproxen**	Arginine-Pluronic f 127	Y	[44]
**Naproxen**	Arginine-Polyoxyethylene (40) stearate	Y	[44]
**Naproxen**	Arginine-Tween 20	n (freeze drying)	[44]
**Naproxen**	Arginine-TPGS 1000	n (freeze drying)	[44]
**Naproxen**	Lysine-Sodium dodecyl sulfate	y	[44]
**Naproxen**	Lysine-Pluronic f 127	y	[44]
**Naproxen**	Lysine-Polyoxyyethylene (40) stearate	y	[44]
**Naproxen**	Lysine-Tween 20	y	[44]
**Naproxen**	Lysine-TPGS 1000	n (freeze drying)	[44]
**Nitrofurantoin**	Arginine	y	[14]
**Nitrofurantoin**	Citrulline	y	[14]
**Ofloxacin**	Tryptophan	y	[46]
**Piroxicam**	Aspartic acid	n (ball millng)	[41]
**Piroxicam**	Phenylalanine	n (ball milling)	[41]
**Piroxicam**	Aspartic acid-Phenylalanine	n (ball milling)	[41]
**Simvastatin**	Lysine-Threonine	n (ball milling)	[18]
**Simvastatin**	Arginine	n (ball milling)	[3–5]
**Simvastatin**	Aspartic acid	n (ball milling)	[3–5,18]
**Simvastatin**	Cysteine	n (ball milling)	[3–5]
**Simvastatin**	Glutamic acid	n (ball milling)	[3–5]
**Simvastatin**	Glycine	n (ball milling)	[3–5]
**Simvastatin**	Histidine	n (ball milling)	[3–5]
**Simvastatin**	Lysine	y	[3–5,7,18,19,47,48]
**Simvastatin**	Proline	n (ball milling)	[3–5]
**Simvastatin**	Phenylalanine	y	[3–5,7]
**Simvastatin**	Tryptophan	depends	[3–5,7,47]
**Simvastatin**	Alanine	n (ball milling)	[3–5]
**Simvastatin**	Asparagine	n (ball milling)	[3–5]
**Simvastatin**	Glutamine	n (ball milling)	[3–5]
**Simvastatin**	Isoleucine	n (ball milling)	[3–5]
**Simvastatin**	Leucine	n (ball milling)	[3–5,47]
**Simvastatin**	Methionine	n (ball milling)	[3–5]
**Simvastatin**	Serine	n (ball milling)	[3–5,18]
**Simvastatin**	Threonine	n (ball milling)	[3–5,18]
**Simvastatin**	Tyrosine	n (ball milling)	[3–5]

**Simvastatin**	Valine	n (ball milling)	[3–5]
**Tadalafil**	Aspartic acid	n (ball milling)	[41]
**Tadalafil**	Phenylalanine	n (ball milling)	[41]
**Tadalafil**	Aspartic acid-Phenylalanine	n (ball milling)	[41]
**Valsartan**	Histidine	y	[49]
**Valsartan**	Arginine	y	[49]
**Valsartan**	Lysine	y	[49]
**Valsartan**	Histidine-Arginine	y	[49]
**Valsartan**	Histidine-Lysine	y	[49]
**Valsartan**	Arginine-Lysine	y	[49]

References

1. Lu, W.; Rades, T.; Rantanen, J.; Yang, M. Inhalable co-amorphous budesonide-arginine dry powders prepared by spray drying. *Int. J. Pharm.* **2019**, *565*, 1-8.
2. Löbmann, K.; Grohganz, H.; Laitinen, R.; Strachan, C.; Rades, T. Amino acids as co-amorphous stabilizers for poorly water soluble drugs--Part 1: preparation, stability and dissolution enhancement. *Eur. J. Pharm. Biopharm.* **2013**, *85*, 873-881.
3. Kasten, G.; Grohganz, H.; Rades, T.; Löbmann, K. Development of a screening method for co-amorphous formulations of drugs and amino acids. *Eur. J. Pharm. Sci.* **2016**, *95*, 28-35.
4. Meng-Lund, H.; Kasten, G.; Jensen, K.T.; Poso, A.; Pantsar, T.; Rades, T.; Rantanen, J.; Grohganz, H. The use of molecular descriptors in the development of co-amorphous formulations. *Eur. J. Pharm. Sci.* **2018**, *119*, 31-38.
5. Chambers, L.I.; Grohganz, H.; Palmelund, H.; Löbmann, K.; Rades, T.; Musa, O.M.; Steed, J.W. Predictive identification of co-formers in co-amorphous systems. *Eur. J. Pharm. Sci.* **2021**, *157*, 105636-105643.
6. Ueda, H.; Wu, W.; Löbmann, K.; Grohganz, H.; Mullertz, A.; Rades, T. Application of a salt coformer in a co-amorphous drug system dramatically enhances the glass transition temperature: a case study of the ternary system carbamazepine, citric acid, and l-arginine. *Mol. Pharm.* **2018**, *15*, 2036-2044.
7. Kasten, G.; Löbmann, K.; Grohganz, H.; Rades, T. Co-former selection for co-amorphous drug-amino acid formulations. *Int. J. Pharm.* **2019**, *557*, 366-373.
8. Mishra, J.; Löbmann, K.; Grohganz, H.; Rades, T. Influence of preparation technique on co-amorphization of carvedilol with acidic amino acids. *Int. J. Pharm.* **2018**, *552*, 407-413.
9. Petry, I.; Löbmann, K.; Grohganz, H.; Rades, T.; Leopold, C.S. In situ co-amorphisation in coated tablets - the combination of carvedilol with aspartic acid during immersion in an acidic medium. *Int. J. Pharm.* **2019**, *558*, 357-366.
10. Influence of polymer addition on the amorphization, dissolution and physical stability of co-amorphous systems. *Int. J. Pharm.* **2020**, 588, 119768-119776.
11. Liu, J.; Rades, T.; Grohganz, H. Determination of the Optimal Molar Ratio in Amino Acid-Based Coamorphous Systems. *Mol Pharm* **2020**, *17*, 1335-1342.
12. Kissi, E.; Kasten, G.; Löbmann, K.; Rades, T.; Grohganz, H. The role of glass transition temperatures in co-amorphous drug-amino acid formulations. *Mol. Pharm.* **2018**, *15*, 4247-4256.
13. Sterren, V.B.; Aiassa, V.; Garnero, C.; Linck, Y.G.; Chattah, A.K.; Monti, G.A.; Longhi, M.R.; Zoppi, A. Preparation of chloramphenicol/amino acid combinations exhibiting enhanced dissolution rates and reduced drug-induced oxidative stress. *AAPS PharmSciTech* **2017**, *18*, 2910-2918.
14. Wu, W.; Löbmann, K.; Rades, T.; Grohganz, H. On the role of salt formation and structural similarity of co-formers in co-amorphous drug delivery systems. *Int J Pharm* **2018**, *535*, 86-94.
15. Petry, I.; Löbmann, K.; Grohganz, H.; Rades, T.; Leopold, C.S. In situ co-amorphisation of arginine with indomethacin or furosemide during immersion in an acidic medium - a proof of concept study. *Eur. J. Pharm. Biopharm.* **2018**, *133*, 151-160.
16. Jensen, K.T.; Larsen, F.H.; Löbmann, K.; Rades, T.; Grohganz, H. Influence of variation in molar ratio on co-amorphous drug-amino acid systems. *Eur. J. Pharm. Biopharm.* **2016**, *107*, 32-39.
17. Jensen, K.T.; Larsen, F.H.; Cornett, C.; Löbmann, K.; Grohganz, H.; Rades, T. Formation mechanism of coamorphous drug-amino acid mixtures. *Mol. Pharm.* **2015**, *12*, 2484-2492.
18. Laitinen, R.; Löbmann, K.; Grohganz, H.; Strachan, C.; Rades, T. Amino acids as co-amorphous excipients for simvastatin and glibenclamide: physical properties and stability. *Mol. Pharm.* **2014**, *11*, 2381-2389.
19. Heikkinen, A.; DeClerck, L.; Löbmann, K.; Grohganz, H.; Rades, T.; Laitinen, R. Dissolution properties of co-amorphous drug-amino acid formulations in buffer and biorelevant media. *Pharmazie* **2015**, *70*, 452-457.
20. Sormunen, H.; Ruponen, M.; Laitinen, R. The effect of co-amorphization of glibenclamide on its dissolution properties and permeability through an MDCKII-MDR1 cell layer. *Int. J. Pharm.* **2019**, *570*, 118653.
21. Ruponen, M.; Visti, M.; Ojarinta, R.; Laitinen, R. Permeability of glibenclamide through a PAMPA membrane: The effect of co-amorphization. *Eur. J. Pharm. Biopharm.* **2018**, *129*, 247-256.

22. Park, H.; Jin Seo, H.; Hong, S.H.; Ha, E.S.; Lee, S.; Kim, J.S.; Baek, I.H.; Kim, M.S.; Hwang, S.J. Characterization and therapeutic efficacy evaluation of glimepiride and L-arginine co-amorphous formulation prepared by supercritical antisolvent process: Influence of molar ratio and preparation methods. *Int. J. Pharm.* **2020**, *581*, 119232.
23. Franca, M.T.; Marcos, T.M.; Pereira, R.N.; Stulzer, H.K. Could the small molecules such as amino acids improve aqueous solubility and stabilize amorphous systems containing Griseofulvin? *Eur J Pharm Sci* **2020**, *143*, 105178.
24. Ruponen, M.; Rusanen, H.; Laitinen, R. Dissolution and permeability properties of co-amorphous formulations of hydrochlorothiazide. *J. Pharm. Sci.* **2020**, *109*, 2252-2261.
25. Zhang, M.; Suo, Z.; Peng, X.; Gan, N.; Zhao, L.; Tang, P.; Wei, X.; Li, H. Microcrystalline cellulose as an effective crystal growth inhibitor for the ternary Ibrutinib formulation. *Carbohydr. Polym.* **2020**, *229*, 115476.
26. Ojarinta, R.; Lerminiaux, L.; Laitinen, R. Spray drying of poorly soluble drugs from aqueous arginine solution. *Int. J. Pharm.* **2017**, *532*, 289-298.
27. Ojarinta, R.; Saarinen, J.; Strachan, C.J.; Korhonen, O.; Laitinen, R. Preparation and characterization of multi-component tablets containing co-amorphous salts: Combining multimodal non-linear optical imaging with established analytical methods. *Eur. J. Pharm. Biopharm.* **2018**, *132*, 112-126.
28. Lenz, E.; Löbmann, K.; Rades, T.; Knop, K.; Kleinebudde, P. Hot melt extrusion and spray drying of co-amorphous indomethacin-arginine with polymers. *J. Pharm. Sci.* **2017**, *106*, 302-312.
29. Ojarinta, R.; Heikkinen, A.T.; Sievanen, E.; Laitinen, R. Dissolution behavior of co-amorphous amino acid-indomethacin mixtures: The ability of amino acids to stabilize the supersaturated state of indomethacin. *Eur. J. Pharm. Biopharm.* **2017**, *112*, 85-95.
30. Petry, I.; Löbmann, K.; Grohganz, H.; Rades, T.; Leopold, C.S. Solid state properties and drug release behavior of co-amorphous indomethacin-arginine tablets coated with Kollicoat® Protect. *Eur. J. Pharm. Biopharm.* **2017**, *119*, 150-160.
31. Mishra, J.; Rades, T.; Löbmann, K.; Grohganz, H. Influence of solvent composition on the performance of spray-dried co-amorphous formulations. *Pharmaceutics* **2018**, *10*.
32. Petry, I.; Löbmann, K.; Grohganz, H.; Rades, T.; Leopold, C.S. Undesired co-amorphisation of indomethacin and arginine during combined storage at high humidity conditions. *Int. J. Pharm.* **2018**, *544*, 172-180.
33. Lenz, E.; Jensen, K.T.; Blaabjerg, L.I.; Knop, K.; Grohganz, H.; Löbmann, K.; Rades, T.; Kleinebudde, P. Solid-state properties and dissolution behaviour of tablets containing co-amorphous indomethacin-arginine. *Eur. J. Pharm. Biopharm.* **2015**, *96*, 44-52.
34. Wickstrom, H.; Palo, M.; Rijckaert, K.; Kolakovic, R.; Nyman, J.O.; Maattanen, A.; Ihalainen, P.; Peltonen, J.; Genina, N.; de Beer, T., et al. Improvement of dissolution rate of indomethacin by inkjet printing. *Eur. J. Pharm. Sci.* **2015**, *75*, 91-100.
35. Jensen, K.T.; Blaabjerg, L.I.; Lenz, E.; Bohr, A.; Grohganz, H.; Kleinebudde, P.; Rades, T.; Lobmann, K. Preparation and characterization of spray-dried co-amorphous drug-amino acid salts. *J. Pharm. Pharmacol.* **2016**, *68*, 615-624.
36. Kasten, G.; Nouri, K.; Grohganz, H.; Rades, T.; Löbmann, K. Performance comparison between crystalline and co-amorphous salts of indomethacin-lysine. *Int. J. Pharm.* **2017**, *533*, 138-144.
37. Walker, G.; Romann, P.; Poller, B.; Löbmann, K.; Grohganz, H.; Rooney, J.S.; Huff, G.S.; Smith, G.P.S.; Rades, T.; Gordon, K.C., et al. Probing pharmaceutical mixtures during milling: the potency of low-frequency raman spectroscopy in identifying disorder. *Mol. Pharm.* **2017**, *14*, 4675-4684.
38. Adhikari, B.R.; Berzins, K.; Fraser-Miller, S.J.; Gordon, K.C.; Das, S.C. Co-amorphization of kanamycin with amino acids improves aerosolization. *Pharmaceutics* **2020**, *12*.
39. Heng, W.; Su, M.; Cheng, H.; Shen, P.; Liang, S.; Zhang, L.; Wei, Y.; Gao, Y.; Zhang, J.; Qian, S. Incorporation of complexation into a coamorphous system dramatically enhances dissolution and eliminates gelation of amorphous lurasidone hydrochloride. *Mol. Pharm.* **2020**, *17*, 84-97.
40. Wu, W.; Grohganz, H.; Rades, T.; Löbmann, K. Comparison of co-former performance in co-amorphous formulations: single amino acids, amino acid physical mixtures, amino acid salts and dipeptides as co-formers. *Eur. J. Pharm. Sci.* **2020**, *156*, 105582-105589.
41. Wu, W.; Löbmann, K.; Schnitzkewitz, J.; Knuhtsen, A.; Pedersen, D.S.; Grohganz, H.; Rades, T. Aspartame as a co-former in co-amorphous systems. *Int. J. Pharm.* **2018**, *549*, 380-387.
42. Wu, W.; Löbmann, K.; Schnitzkewitz, J.; Knuhtsen, A.; Pedersen, D.S.; Rades, T.; Grohganz, H. Dipeptides as co-formers in co-amorphous systems. *Eur. J. Pharm. Biopharm.* **2019**, *134*, 68-76.
43. Jensen, K.T.; Löbmann, K.; Rades, T.; Grohganz, H. Improving co-amorphous drug formulations by the addition of the highly water soluble amino acid, proline. *Pharmaceutics* **2014**, *6*, 416-435.
44. Wostry, M.; Plappert, H.; Grohganz, H. Preparation of co-amorphous systems by freeze-drying. *Pharmaceutics* **2020**, *12*.
45. Kasten, G.; Lobo, L.; Dengale, S.; Grohganz, H.; Rades, T.; Lobmann, K. In vitro and in vivo comparison between crystalline and co-amorphous salts of naproxen-arginine. *Eur. J. Pharm. Biopharm.* **2018**, *132*, 192-199.
46. Zhu, S.; Gao, H.; Babu, S.; Garad, S. Co-amorphous formation of high-dose zwitterionic compounds with amino acids to improve solubility and enable parenteral delivery. *Mol. Pharm.* **2018**, *15*, 97-107.
47. Lu, W.; Rades, T.; Rantanen, J.; Chan, H.K.; Yang, M. Amino acids as stabilizers for spray-dried simvastatin powder for inhalation. *Int. J. Pharm.* **2019**, *572*, 118724.

48. Craye, G.; Lobmann, K.; Grohganz, H.; Rades, T.; Laitinen, R. Characterization of amorphous and co-amorphous simvastatin formulations prepared by spray drying. *Molecules* **2015**, *20*.
49. Huang, Y.; Zhang, Q.; Wang, J.R.; Lin, K.L.; Mei, X. Amino acids as co-amorphous excipients for tackling the poor aqueous solubility of valsartan. *Pharm. Dev. Technol.* **2017**, *22*, 69-76.

Table S2. Overview of screened CAMS and non-CAMS with a drug as a co-former. (y: yes. n: no. depends: dependent on the preparation method and/or the preparation parameters. -: unknown).

Drug 1	Drug 2	CAMS (y/n/depends)	Reference
**Aceclofenac**	Naproxen	y	[1]
**Amoxicillin**	Omeprazole	y	[2]
**Atenolol**	Hydrochlorothiazide	depends	[3,4]
**Atorvastatin**	Carvedilol	y	[5]
**Atorvastatin**	Glibenclamide	y	[5]
**Atorvastatin**	Naringin	y	[6]
**Budesonide**	Theophylline	y	[7]
**Cimetidine**	Salycilamide	n (solvent evaporation)	[8]
**Cimetidine**	Phenacetin	n (solvent evaporation)	[8]
**Cimetidine**	Diflusinal	Y	[9]
**Cimetidine**	Piroxicam	y	[10,11]
**Clotrimazole**	Carvedilol	y	[12]
**Clotrimazole**	Indomethacin	y	[12]
**Curcumin**	Piperine	y	[13]
**Curcumin**	Artemisinin	y	[14,15]
**Docetaxel**	Bicalutamide	y	[16]
**Docetaxel**	Myricetin	y	[17]
**Ezetimib**	Indapamide	y	[18]
**Ezetimib**	Lovastatin	y	[19]
**Ezetimib**	Simvastatin	y	[20,21]
**Famotidine**	Ibuprofen	y	[22]
**Febuxostat**	Indomethacin	y	[23]
**Felbinac**	Naproxen	n (melt-quench)	[1]
**Flutamide**	Bicalutamide	y	[24]
**Gliclazide**	Chlorothiazide	y	[25]
**Gliclazide**	Hydrochlorothiazide	y	[25]
**Gliclazide**	Indapamide	y	[25]
**Gliclazide**	Triamterene	y	[25]
**Gliclazide**	Nifedipine	y	[25]
**Gliclazide**	Benzamidine	y	[25]
**Glipizide**	Atorvastatin	y	[26]
**Ibuprofen**	Naproxen	n (melt-quench)	[1]
**Indomethacin**	Naproxen	y	[27–30] [1,31–35]
**Indomethacin**	Acetylsalicylacid	y	[36]
**Indomethacin**	Paracetamol	y	[32,33,36,37]
**Indomethacin**	Glibenclamide	y	[36]
**Indomethacin**	Cimetidine	y	[8,32,33,35,38–40]
**Indomethacin**	Ranitidine hydrochloride	y	[41]
**Indomethacin**	Antipyrine	-	[42]
**Indomethacin**	Felodipine	y	[12]
**Indomethacin**	Cimetidine	y	[12]
**Indomethacin**	Lidocaine	y	[43]
**Indomethacin**	Lidocaine hydrochloride	y	[44]

**Indomethacin**	Nicotinamide	y	[45]
**Irbesartan**	Atenolol	y	[4]
**Irbesartan**	Glimepiride	y	[46]
**Itraconazole**	Indomethacin	y	[12]
**Itraconazole**	Clotrimazole	y	[12]
**Itraconazole**	Cimetidine	y	[12]
**Itraconazole**	Carvedilol	y	[12]
**Ketoprofen**	Naproxen	y	[1]
**Lacidipine**	Spironolactone	y	[47]
**Loxoprofen**	Naproxen	y	[1]
**Lurasidone hydrochloride**	Puerarin	y	[48]
**Lurasidone hydrochloride**	Repaglinide	y	[49]
**Mebendazole**	Ascorbic acid	n (ball milling)	[50]
**Mebendazole**	Caffeine	n (ball milling)	[50]
**Mebendazole**	Catechol	n (ball milling)	[50]
**Mebendazole**	Flurbiprofen	y	[50]
**Mebendazole**	Ketoprofen	y	[50]
**Mebendazole**	Piracetam	n (ball milling)	[50]
**Mebendazole**	Theophylline	y	[50]
**Naproxen**	Cimetidine	y	[32,33,35,39,51,52]
**Naproxen**	Naproxen sodium	y	[1]
**Naproxen sodium**	Indomethacin	y	[29]
**Nateglinide**	Metformin hydrochloride	y	[53,54]
**Nifedipine**	Cimetidine	y	[55]
**Nifedipine**	Nimodipine	y	[56]
**Nifedipine**	Paracetamol	y	[34]
**Nifedipine**	Naproxen	y	[12]
**Nifedipine**	Loratadine	y	[12]
**Nifedipine**	Indomethacin	y	[12]
**Nifedipine**	Itraconazole	y	[12]
**Nifedipine**	Salicin	y	[12]
**Nimesulide**	Carvedilol	y	[55]
**Nimesulide**	Indomethacin	y	[57]
**Olmesartan medoxomil**	Hydrochlorothiazide	y	[58]
**Paracetamol**	Antipyrine	y	[59]
**Paracetamol**	Glibenclamide	y	[36]
**Paracetamol**	Acetylsalicylacid	y	[36]
**Paracetamol**	Celecoxib	y	[34]
**Phenobarbital**	Salicin	-	[42]
**Phenobarbital**	Antipyrine	-	[42]
**Probucol**	Nifedipine	y	[12]
**Probucol**	Itraconazole	y	[12]
**Probucol**	Salicin	n (melt-quench)	[12]
**Probucol**	Clotrimazole	y	[12]
**Probucol**	Indomethacin	y	[12]
**Probucol**	Carvedilol	n (melt-quench)	[12]
**Probucol**	Cimetidine	n (melt-quench)	[12]
**Quercetin**	Ritonavir	y	[60]
**Ritonavir**	Indomethacin	y	[61]
**Ritonavir**	Lopinavir	y	[62]
**Sacubitril**	Valsartan	y	[63]

**Simvastatin**	Glipizide	y	[64]
**Simvastatin**	Nifedipine	y	[65]
**Talinolol**	Naringin	y	[66]
**Telmisaratan**	Atenolol	n (co-grinding)	[4]
**Terfenadine**	Paracetamol	y	[36,37]
**Terfenadine**	Indomethacin	y	[32,36,37]
**Terfenadine**	Acetylsalicylacid	y	[36]
**Tranilast**	Diphenhydramine hydrochloride	y	[67]
**Valsartan**	Nifedipine	y	[68]

References

1. Ueda, H.; Muranushi, N.; Sakuma, S.; Ida, Y.; Endoh, T.; Kadota, K.; Tozuka, Y. A Strategy for Co-former Selection to Design Stable Co-amorphous Formations Based on Physicochemical Properties of Non-steroidal Inflammatory Drugs. *Pharm. Res.* **2016**, *33*, 1018-1029, doi:10.1007/s11095-015-1848-2.
2. Russo, M.G.; Sancho, M.I.; Silva, L.M.; Baldoni, H.A.; Venancio, T.; Ellena, J.; Narda, G.E. Looking for the interactions between omeprazole and amoxicillin in a disordered phase. An experimental and theoretical study. *Spectrochim. Acta. A Mol. Biomol. Spectrosc.* **2016**, *156*, 70-77, doi:10.1016/j.saa.2015.11.021.
3. Moinuddin, S.M.; Ruan, S.; Huang, Y.; Gao, Q.; Shi, Q.; Cai, B.; Cai, T. Facile formation of co-amorphous atenolol and hydrochlorothiazide mixtures via cryogenic-milling: Enhanced physical stability, dissolution and pharmacokinetic profile. *Int J Pharm* **2017**, *532*, 393-400, doi:10.1016/j.ijpharm.2017.09.020.
4. Haneef, J.; Chadha, R. Drug-Drug Multicomponent Solid Forms: Cocrystal, Coamorphous and Eutectic of Three Poorly Soluble Antihypertensive Drugs Using Mechanochemical Approach. *AAPS PharmSciTech* **2017**, *18*, 2279-2290, doi:10.1208/s12249-016-0701-1.
5. Shayanfar, A.; Jouyban, A. Drug–Drug Coamorphous Systems: Characterization and Physicochemical Properties of Coamorphous Atorvastatin with Carvedilol and Glibenclamide. *Journal of Pharmaceutical Innovation* **2013**, *8*, 218-228, doi:10.1007/s12247-013-9162-1.
6. Nair, A.; Varma, R.; Gourishetti, K.; Bhat, K.; Dengale, S. Influence of Preparation Methods on Physicochemical and Pharmacokinetic Properties of Co-amorphous Formulations: The Case of Co-amorphous Atorvastatin: Naringin. *Journal of Pharmaceutical Innovation* **2019**, *15*, 365-379, doi:10.1007/s12247-019-09381-9.
7. Leng, D.; Kissi, E.O.; Lobmann, K.; Thanki, K.; Fattal, E.; Rades, T.; Foged, C.; Yang, M. Design of Inhalable Solid Dosage Forms of Budesonide and Theophylline for Pulmonary Combination Therapy. *AAPS PharmSciTech* **2019**, *20*, 137, doi:10.1208/s12249-019-1344-9.
8. Yamamura, S.; Gotoh, H.; Sakamoto, Y.; Momose, Y. Physicochemical properties of amorphous precipitates of cimetidine–indomethacin binary system. *European journal of pharmaceutics and biopharmaceutics* **2000**, *49*, 259-265.
9. Yamamura, S.; Gotoh, H.; Sakamoto, Y.; Momose, Y. Physicochemical properties of amorphous salt of cimetidine and diflunisal system. *Int. J. Pharm.* **2002**, *241*, 213-221.
10. Tantishaiyakul, V.; Songkro, S.; Suknuntha, K.; Permkum, P.; Pipatwarakul, P. Crystal structure transformations and dissolution studies of cimetidine-piroxicam coprecipitates and physical mixtures. *AAPS PharmSciTech* **2009**, *10*, 789-795, doi:10.1208/s12249-009-9263-9.
11. Tantishaiyakul, V.; Suknuntha, K.; Vao-Soongnern, V. Characterization of cimetidine-piroxicam coprecipitate interaction using experimental studies and molecular dynamic simulations. *AAPS PharmSciTech* **2010**, *11*, 952-958, doi:10.1208/s12249-010-9461-5.
12. Mizoguchi, R.; Waraya, H.; Hirakura, Y. Application of Co-Amorphous Technology for Improving the Physicochemical Properties of Amorphous Formulations. *Mol Pharm* **2019**, *16*, 2142-2152, doi:10.1021/acs.molpharmaceut.9b00105.
13. Wang, R.; Han, J.; Jiang, A.; Huang, R.; Fu, T.; Wang, L.; Zheng, Q.; Li, W.; Li, J. Involvement of metabolism-permeability in enhancing the oral bioavailability of curcumin in excipient-free solid dispersions co-formed with piperine. *Int J Pharm* **2019**, *561*, 9-18, doi:10.1016/j.ijpharm.2019.02.027.
14. Suresh, K.; Mannava, M.K.C.; Nangia, A. A novel curcumin–artemisinin coamorphous solid: physical properties and pharmacokinetic profile. *RSC Adv.* **2014**, *4*, 58357-58361, doi:10.1039/c4ra11935e.
15. Mannava, M.K.C.; Suresh, K.; Kumar Bommaka, M.; Bhavani Konga, D.; Nangia, A. Curcumin-Artemisinin Coamorphous Solid: Xenograft Model Preclinical Study. *Pharmaceutics* **2018**, *10*, doi:10.3390/pharmaceutics10010007.
16. Bohr, A.; Nascimento, T.L.; Harmankaya, N.; Weisser, J.J.; Wang, Y.; Grohganz, H.; Rades, T.; Lobmann, K. Efflux Inhibitor Bicalutamide Increases Oral Bioavailability of the Poorly Soluble Efflux Substrate Docetaxel in Co-Amorphous Anti-Cancer Combination Therapy. *Molecules* **2019**, *24*, doi:10.3390/molecules24020266.
17. Wei, Y.; Zhou, S.; Hao, T.; Zhang, J.; Gao, Y.; Qian, S. Further enhanced dissolution and oral bioavailability of docetaxel by coamorphization with a natural P-gp inhibitor myricetin. *Eur J Pharm Sci* **2019**, *129*, 21-30, doi:10.1016/j.ejps.2018.12.016.

18. Knapik, J.; Wojnarowska, Z.; Grzybowska, K.; Jurkiewicz, K.; Tajber, L.; Paluch, M. Molecular Dynamics and Physical Stability of Coamorphous Ezetimib and Indapamide Mixtures. *Mol Pharm* **2015**, *12*, 3610-3619, doi:10.1021/acs.molpharmaceut.5b00334.
19. Riekes, M.K.; Engelen, A.; Appeltans, B.; Rombaut, P.; Stulzer, H.K.; Van den Mooter, G. New Perspectives for Fixed Dose Combinations of Poorly Water-Soluble Compounds: a Case Study with Ezetimibe and Lovastatin. *Pharm. Res.* **2016**, *33*, 1259-1275, doi:10.1007/s11095-016-1870-z.
20. Knapik-Kowalczuk, J.; Chmiel, K.; Jurkiewicz, K.; Correia, N.T.; Sawicki, W.; Paluch, M. Physical Stability and Viscoelastic Properties of Co-Amorphous Ezetimibe/Simvastatin System. *Pharmaceuticals (Basel)* **2019**, *12*, doi:10.3390/ph12010040.
21. Phan, A.D.; Knapik-Kowalczuk, J.; Paluch, M.; Hoang, T.X.; Wakabayashi, K. Theoretical Model for the Structural Relaxation Time in Coamorphous Drugs. *Mol Pharm* **2019**, *16*, 2992-2998, doi:10.1021/acs.molpharmaceut.9b00230.
22. Russo, M.G.; Baldoni, H.A.; Davila, Y.A.; Brusau, E.V.; Ellena, J.A.; Narda, G.E. Rational Design of a Famotidine-Ibuprofen Coamorphous System: An Experimental and Theoretical Study. *J Phys Chem B* **2018**, *122*, 8772-8782, doi:10.1021/acs.jpcb.8b06105.
23. Moinuddin, S.M.; Shi, Q.; Tao, J.; Guo, M.; Zhang, J.; Xue, Q.; Ruan, S.; Cai, T. Enhanced Physical Stability and Synchronized Release of Febuxostat and Indomethacin in Coamorphous Solids. *AAPS PharmSciTech* **2020**, *21*, 41, doi:10.1208/s12249-019-1578-6.
24. Pacult, J.; Rams-Baron, M.; Chmiel, K.; Jurkiewicz, K.; Antosik, A.; Szafraniec, J.; Kurek, M.; Jachowicz, R.; Paluch, M. How can we improve the physical stability of co-amorphous system containing flutamide and bicalutamide? The case of ternary amorphous solid dispersions. *Eur J Pharm Sci* **2019**, *136*, 104947, doi:10.1016/j.ejps.2019.06.001.
25. Aljohani, M.; MacFhionnghaile, P.; McArdle, P.; Erxleben, A. Investigation of the formation of drug-drug cocrystals and coamorphous systems of the antidiabetic drug gliclazide. *Int J Pharm* **2019**, *561*, 35-42, doi:10.1016/j.ijpharm.2019.02.024.
26. Renuka; Singh, S.K.; Gulati, M.; Narang, R. Stable amorphous binary systems of glipizide and atorvastatin powders with enhanced dissolution profiles: formulation and characterization. *Pharm Dev Technol* **2017**, *22*, 13-25, doi:10.3109/10837450.2015.1125921.
27. Beyer, A.; Grohganz, H.; Lobmann, K.; Rades, T.; Leopold, C.S. Multivariate Quantification of the Solid State Phase Composition of Co-Amorphous Naproxen-Indomethacin. *Molecules* **2015**, *20*, 19571-19587, doi:10.3390/molecules201019571.
28. Beyer, A.; Grohganz, H.; Lobmann, K.; Rades, T.; Leopold, C.S. Influence of the cooling rate and the blend ratio on the physical stability of co-amorphous naproxen/indomethacin. *Eur J Pharm Biopharm* **2016**, *109*, 140-148, doi:10.1016/j.ejpb.2016.10.002.
29. Beyer, A.; Grohganz, H.; Lobmann, K.; Rades, T.; Leopold, C.S. Improvement of the physicochemical properties of Co-amorphous naproxen-indomethacin by naproxen-sodium. *Int J Pharm* **2017**, *526*, 88-94, doi:10.1016/j.ijpharm.2017.04.011.
30. Beyer, A.; Radi, L.; Grohganz, H.; Lobmann, K.; Rades, T.; Leopold, C.S. Preparation and recrystallization behavior of spray-dried co-amorphous naproxen-indomethacin. *Eur J Pharm Biopharm* **2016**, *104*, 72-81, doi:10.1016/j.ejpb.2016.04.019.
31. Löbmann, K.; Laitinen, R.; Grohganz, H.; Gordon, K.C.; Strachan, C.; Rades, T. Coamorphous Drug Systems: Enhanced Physical Stability and Dissolution Rate of Indomethacin and Naproxen. *Molecular Pharmaceutics* **2011**, *8*, 1919-1928, doi:10.1021/mp2002973.
32. Pajula, K.; Hyyrylainen, J.; Koistinen, A.; Leskinen, J.T.T.; Korhonen, O. Detection of amorphous-amorphous phase separation in small molecular co-amorphous mixtures with SEM-EDS. *Eur J Pharm Biopharm* **2020**, *150*, 43-49, doi:10.1016/j.ejpb.2020.03.002.
33. Chieng, N.; Teo, X.; Cheah, M.H.; Choo, M.L.; Chung, J.; Hew, T.K.; Keng, P.S. Molecular Dynamics and Physical Stability of Pharmaceutical Co-amorphous Systems: Correlation Between Structural Relaxation Times Measured by Kohlrausch-Williams-Watts With the Width of the Glass Transition Temperature (DeltaTg) and the Onset of Crystallization. *J. Pharm. Sci.* **2019**, *108*, 3848-3858, doi:10.1016/j.xphs.2019.09.013.
34. Kissi, E.O.; Khorami, K.; Rades, T. Determination of Stable Co-Amorphous Drug-Drug Ratios from the Eutectic Behavior of Crystalline Physical Mixtures. *Pharmaceutics* **2019**, *11*, doi:10.3390/pharmaceutics11120628.
35. Liu, W.; Liu, Y.; Huang, J.; Lin, Z.; Pan, X.; Zeng, X.; Lamy de la Chapelle, M.; Zhang, Y.; Fu, W. Identification and investigation of the vibrational properties of crystalline and co-amorphous drugs with Raman and terahertz spectroscopy. *Biomed Opt Express* **2019**, *10*, 4290-4304, doi:10.1364/BOE.10.004290.
36. Pajula, K.; Wittoek, L.; Lehto, V.P.; Ketolainen, J.; Korhonen, O. Phase separation in coamorphous systems: in silico prediction and the experimental challenge of detection. *Mol Pharm* **2014**, *11*, 2271-2279, doi:10.1021/mp400712m.
37. Kilpelainen, T.; Pajula, K.; Ervasti, T.; Uurasjarvi, E.; Koistinen, A.; Korhonen, O. Raman imaging of amorphous-amorphous phase separation in small molecule co-amorphous systems. *Eur J Pharm Biopharm* **2020**, *155*, 49-54, doi:10.1016/j.ejpb.2020.08.007.
38. Otsuka, Y.; Kuwashima, W.; Tanaka, Y.; Yamaki, Y.; Shimada, Y.; Goto, S. Effects of Heat Treatment on Indomethacin-Cimetidine Mixture; Investigation of Drug-Drug Interaction Using Singular Value Decomposition in FTIR Spectroscopy. *J. Pharm. Sci.* **2020**, 10.1016/j.xphs.2020.09.049, doi:10.1016/j.xphs.2020.09.049.
39. Lim, A.W.; Lobmann, K.; Grohganz, H.; Rades, T.; Chieng, N. Investigation of physical properties and stability of indomethacin-cimetidine and naproxen-cimetidine co-amorphous systems prepared by quench cooling, coprecipitation and ball milling. *J Pharm Pharmacol* **2016**, *68*, 36-45, doi:10.1111/jphp.12494.

40. Arnfast, L.; Kamruzzaman, M.; Lobmann, K.; Aho, J.; Baldursdottir, S.; Rades, T.; Rantanen, J. Melt Extrusion of High-Dose Co-Amorphous Drug-Drug Combinations : Theme: Formulation and Manufacturing of Solid Dosage Forms Guest Editors: Tony Zhou and Tonglei Li. *Pharm. Res.* **2017**, *34*, 2689-2697, doi:10.1007/s11095-017-2254-8.
41. Chieng, N.; Aaltonen, J.; Saville, D.; Rades, T. Physical characterization and stability of amorphous indomethacin and ranitidine hydrochloride binary systems prepared by mechanical activation. *Eur J Pharm Biopharm* **2009**, *71*, 47-54, doi:10.1016/j.ejpb.2008.06.022.
42. FUKUOKA, E.; MAKITA, M.; YAMAMURA, S. Glassy state of pharmaceuticals. III.: Thermal properties and stability of glassy pharmaceuticals and their binary glass systems. *Chemical and pharmaceutical bulletin* **1989**, *37*, 1047-1050.
43. Shimada, Y.; Goto, S.; Uchiro, H.; Hirabayashi, H.; Yamaguchi, K.; Hirota, K.; Terada, H. Features of heat-induced amorphous complex between indomethacin and lidocaine. *Colloids Surf. B. Biointerfaces* **2013**, *102*, 590-596, doi:10.1016/j.colsurfb.2012.08.060.
44. Shimada, Y.; Goto, S.; Uchiro, H.; Hirota, K.; Terada, H. Characteristics of amorphous complex formed between indomethacin and lidocaine hydrochloride. *Colloids Surf. B. Biointerfaces* **2013**, *105*, 98-105, doi:10.1016/j.colsurfb.2012.12.026.
45. Tawfeek, H.M.; Chavan, T.; Kunda, N.K. Effect of Spray Drying on Amorphization of Indomethacin Nicotinamide Cocrystals; Optimization, Characterization, and Stability Study. *AAPS PharmSciTech* **2020**, *21*, 181, doi:10.1208/s12249-020-01732-x.
46. Cruz-Angeles, J.; Videa, M.; Martinez, L.M. Highly Soluble Glimepiride and Irbesartan Co-amorphous Formulation with Potential Application in Combination Therapy. *AAPS PharmSciTech* **2019**, *20*, 144, doi:10.1208/s12249-019-1359-2.
47. Wang, Z.; Sun, M.; Liu, T.; Gao, Z.; Ye, Q.; Tan, X.; Hou, Y.; Sun, J.; Wang, D.; He, Z. Co-amorphous solid dispersion systems of lacidipine-spironolactone with improved dissolution rate and enhanced physical stability. *Asian J Pharm Sci* **2019**, *14*, 95-103, doi:10.1016/j.ajps.2018.11.001.
48. Wang, S.; Heng, W.; Wang, X.; He, X.; Zhang, Z.; Wei, Y.; Zhang, J.; Gao, Y.; Qian, S. Coamorphization combined with complexation enhances dissolution of lurasidone hydrochloride and puerarin with synchronized release. *Int J Pharm* **2020**, *588*, 119793, doi:10.1016/j.ijpharm.2020.119793.
49. Qian, S.; Li, Z.; Heng, W.; Liang, S.; Ma, D.; Gao, Y.; Zhang, J.; Wei, Y. Charge-assisted intermolecular hydrogen bond formed in coamorphous system is important to relieve the pH-dependent solubility behavior of lurasidone hydrochloride. *RSC Advances* **2016**, *6*, 106396-106412, doi:10.1039/c6ra18022a.
50. Chambers, L.I.; Grohganz, H.; Palmelund, H.; Lobmann, K.; Rades, T.; Musa, O.M.; Steed, J.W. Predictive Identification of Co-formers in Co-amorphous Systems. *Eur J Pharm Sci* **2020**, 10.1016/j.ejps.2020.105636, 105636, doi:10.1016/j.ejps.2020.105636.
51. Alleso, M.; Chieng, N.; Rehder, S.; Rantanen, J.; Rades, T.; Aaltonen, J. Enhanced dissolution rate and synchronized release of drugs in binary systems through formulation: Amorphous naproxen-cimetidine mixtures prepared by mechanical activation. *J Control Release* **2009**, *136*, 45-53, doi:10.1016/j.jconrel.2009.01.027.
52. Yamamura, S.; Momose, Y.; Takahashi, K.; Nagatani, S. Solid-state interaction between cimetidine and naproxen. *Drug Stability* **1996**, *1*, 173-178.
53. Wairkar, S.; Gaud, R. Co-Amorphous Combination of Nateglinide-Metformin Hydrochloride for Dissolution Enhancement. *AAPS PharmSciTech* **2016**, *17*, 673-681, doi:10.1208/s12249-015-0371-4.
54. Wairkar, S.; Gaud, R. Development and Characterization of Microstructured, Spray-Dried Co-Amorphous Mixture of Antidiabetic Agents Stabilized by Silicate. *AAPS PharmSciTech* **2019**, *20*, 141, doi:10.1208/s12249-019-1352-9.
55. Martinez, L.M.; Videa, M.; Sosa, N.G.; Ramirez, J.H.; Castro, S. Long-Term Stability of New Co-Amorphous Drug Binary Systems: Study of Glass Transitions as a Function of Composition and Shelf Time. *Molecules* **2016**, *21*, doi:10.3390/molecules21121712.
56. Knapik-Kowalczuk, J.; Tu, W.; Chmiel, K.; Rams-Baron, M.; Paluch, M. Co-Stabilization of Amorphous Pharmaceuticals-The Case of Nifedipine and Nimodipine. *Mol Pharm* **2018**, *15*, 2455-2465, doi:10.1021/acs.molpharmaceut.8b00308.
57. Wang, M.; Liu, S.; Jia, L.; Zhang, J.; Du, S.; Gong, J. Exploring the physical stability of three nimesulide-indomethacin co-amorphous systems from the perspective of molecular aggregates. *Eur J Pharm Sci* **2020**, *147*, 105294, doi:10.1016/j.ejps.2020.105294.
58. Abdelquader, M.M.; Essa, E.A.; El Maghraby, G.M. Inhibition of Co-Crystallization of Olmesartan Medoxomil and Hydrochlorothiazide for Enhanced Dissolution Rate in Their Fixed Dose Combination. *AAPS PharmSciTech* **2018**, *20*, 3, doi:10.1208/s12249-018-1207-9.
59. Martinez, L.M.; Videa, M.; Lopez-Silva, G.A.; de Los Reyes, C.A.; Cruz-Angeles, J.; Gonzalez, N. Stabilization of amorphous paracetamol based systems using traditional and novel strategies. *Int J Pharm* **2014**, *477*, 294-305, doi:10.1016/j.ijpharm.2014.10.021.
60. Dengale, S.J.; Hussen, S.S.; Krishna, B.S.; Musmade, P.B.; Gautham Shenoy, G.; Bhat, K. Fabrication, solid state characterization and bioavailability assessment of stable binary amorphous phases of Ritonavir with Quercetin. *Eur J Pharm Biopharm* **2015**, *89*, 329-338, doi:10.1016/j.ejpb.2014.12.025.
61. Dengale, S.J.; Ranjan, O.P.; Hussen, S.S.; Krishna, B.S.; Musmade, P.B.; Gautham Shenoy, G.; Bhat, K. Preparation and characterization of co-amorphous Ritonavir-Indomethacin systems by solvent evaporation technique: improved dissolution behavior and physical stability without evidence of intermolecular interactions. *Eur J Pharm Sci* **2014**, *62*, 57-64, doi:10.1016/j.ejps.2014.05.015.

62. Sai Krishna Anand, V.; Sakhare, S.D.; Navya Sree, K.S.; Nair, A.R.; Raghava Varma, K.; Gourishetti, K.; Dengale, S.J. The relevance of co-amorphous formulations to develop supersaturated dosage forms: In-vitro, and ex-vivo investigation of Ritonavir-Lopinavir co-amorphous materials. *Eur J Pharm Sci* **2018**, *123*, 124-134, doi:10.1016/j.ejps.2018.07.046.
63. Zhang, Y.; Gao, Y.; Du, X.; Guan, R.; He, Z.; Liu, H. Combining Co-Amorphous-Based Spray Drying with Inert Carriers to Achieve Improved Bioavailability and Excellent Downstream Manufacturability. *Pharmaceutics* **2020**, *12*, doi:10.3390/pharmaceutics12111063.
64. Lobmann, K.; Strachan, C.; Grohganz, H.; Rades, T.; Korhonen, O.; Laitinen, R. Co-amorphous simvastatin and glipizide combinations show improved physical stability without evidence of intermolecular interactions. *Eur J Pharm Biopharm* **2012**, *81*, 159-169, doi:10.1016/j.ejpb.2012.02.004.
65. Martinez-Jimenez, C.; Cruz-Angeles, J.; Videa, M.; Martinez, L.M. Co-Amorphous Simvastatin-Nifedipine with Enhanced Solubility for Possible Use in Combination Therapy of Hypertension and Hypercholesterolemia. *Molecules* **2018**, *23*, doi:10.3390/molecules23092161.
66. Teja, A.; Musmade, P.B.; Khade, A.B.; Dengale, S.J. Simultaneous improvement of solubility and permeability by fabricating binary glassy materials of Talinolol with Naringin: Solid state characterization, in-vivo in-situ evaluation. *Eur J Pharm Sci* **2015**, *78*, 234-244, doi:10.1016/j.ejps.2015.08.002.
67. Ueda, H.; Kadota, K.; Imono, M.; Ito, T.; Kunita, A.; Tozuka, Y. Co-amorphous Formation Induced by Combination of Tranilast and Diphenhydramine Hydrochloride. *J. Pharm. Sci.* **2017**, *106*, 123-128, doi:10.1016/j.xphs.2016.07.009.
68. Lodagekar, A.; Chavan, R.B.; Mannava, M.K.C.; Yadav, B.; Chella, N.; Nangia, A.K.; Shastri, N.R. Co amorphous valsartan nifedipine system: Preparation, characterization, in vitro and in vivo evaluation. *Eur J Pharm Sci* **2019**, *139*, 105048, doi:10.1016/j.ejps.2019.105048.

Table S3. Overview of screened CAMS and non-CAMS with organic acids as co-formers. (y: yes. n: no. –: unknown).

Drug	Organic Acid	CAMS	Reference
**Acyclovir**	Tartaric acid	n (solvent evaporation)	[1]
**Acyclovir**	Citric acid	y	[1]
**Azelnidipine**	Oxalic acid	y	[2]
**Azelnidipine**	Maleic acid	y	[3]
**Carbamazepine**	Citric acid	y	[4]
**Carbamazepine**	Benzoic acid	y	[5]
**Carbamazepine**	Maleic acid	y	[5]
**Carbamazepine**	Succinic acid	y	[5]
**Carbamazepine**	Tartaric acid	y	[5]
**Carvedilol**	Benzoic acid	y	[6]
**Carvedilol**	Malic acid	y	[6]
**Carvedilol**	Citric acid	y	[6]
**Ciprofloxacin**	Tartaric acid	y	[7]
**Ciprofloxacin**	Succinic acid	y	[8]
**Clozapine**	Tartaric acid	y	[9]
**Clozapine**	Citric acid	y	[9]
**Clozapine**	Oxalic acid	y	[9]
**Curcumin**	Folic acid dihydrate	y	[10]
**Curcumin**	Suberic acid	n (Liquid-assisted grinding)	[10]
**Desloratadine**	Benzoic acid	y	[11]
**Ibrutinib**	Tartaric acid	n (ball milling)	[12]
**Ibrutinib**	Citric acid	y	[12]
**Ibrutinib**	Succinic acid	y	[12]
**Ibrutinib**	Oxalic acid	y	[12]
**Indomethacin**	Citric acid	y	[13]
**Itraconazole**	Fumaric acid	y	[14]
**Itraconazole**	Tartaric acid	y	[14]
**Ketoconazole**	Oxalic acid	y	[15,16]
**Ketoconazole**	Tartaric acid	y	[15,16]
**Ketoconazole**	Citric acid	y	[15,16]

**Ketoconazole**	Succinic acid	y	[15,16]
**Loratadine**	Citric acid	y	[17]
**Mebendazole**	2,4-Dihydroxybenzoic acid	y	[18]
**Mebendazole**	3,5-Dihydroxybenzoic acid	y	[18]
**Mebendazole**	3-Aminobenzoic acid	n (ball milling)	[18]
**Mebendazole**	4-Aminobenzoic acid	y	[18]
**Mebendazole**	4-Aminosalicylic acid	y	[18]
**Mebendazole**	5-Aminosalicylic acid	n (ball milling)	[18]
**Mebendazole**	Fumaric acid	y	[18]
**Mebendazole**	Gallic acid	y	[18]
**Mebendazole**	Glycolic acid	n (ball milling)	[18]
**Mebendazole**	Maleic aicd	y	[18]
**Mebendazole**	Oxalic acid	y	[18]
**Mebendazole**	Succinic acid	n (ball milling)	[18]
**Mebendazole**	Tartaric acid	n (ball milling)	[18]
**Mirabegron**	Fumaric acid	y	[19]
**Mirabegron**	Pyroglutamic acid	y	[19]
**Mirabegron**	Citric acid	y	[19]
**Naproxen**	Flufenamic acid	y	[20]
**Olanzapine**	Ascorbic acid	y	[21]
**Olanzapine**	Citric acid	y	[21]
**Olanzapine**	Tartaric acid	y	[21]
**Paracetamol**	Citric acid	y	[22,23]
**Paracetamol**	Citric acid anhydrate	y	[23]
**Rofecoxib**	Citric acid	-	[24]
**Sulfamerazine**	Citric acid	y	[25]
**Sulfamerazine**	Deoxycholic acid	y	[26]
**Sulfamerazine**	Oxalic acid	y	[27]
**Sulfamerazine**	Tartaric acid	y	[27]
**Sulfamerazine**	Citric acid	y	[26,27]
**Sulfamerazine**	Adipic acid	n (ball milling)	[27]
**Sulfamerazine**	Barbituric acid	y	[27]
**Sulfamerazine**	Fumaric acid	y	[27]
**Sulfamerazine**	Glutaric acid	n (ball milling)	[27]
**Sulfamerazine**	Maleic acid	n (ball milling)	[27]
**Sulfamerazine**	Malonic acid	n (ball milling)	[27]
**Sulfamerazine**	Pimelic acid	n (ball milling)	[27]
**Sulfamerazine**	Succinic acid	n (ball milling)	[27]
**Sulfamerazine**	Terephthalic acid	n (ball milling)	[27]
**Sulfathiazole I**	Oxalic acid	n (ball milling)	[28]
**Sulfathiazole I**	Tartaric acid	y	[28]
**Sulfathiazole I**	Citric acid	y	[28]
**Sulfathiazole I**	Fumaric acid	n (ball milling)	[28]
**Sulfathiazole I**	Malic acid	n (ball milling)	[28]
**Sulfathiazole I**	Malonic acid	n (ball milling)	[28]
**Sulfathiazole I**	Succinic acid	n (ball milling)	[28]
**Sulfathiazole I**	Glutaric acid	n (ball milling)	[28]
**Sulfathiazole I**	Adipic acid	n (ball milling)	[28]
**Sulfathiazole I**	Pimelic acid	n (ball milling)	[28]
**Sulfathiazole III**	Oxalic acid	n (ball milling)	[28]
**Sulfathiazole III**	Tartaric acid	y	[28]

**Sulfathiazole III**	Citric acid	y	[28]
**Sulfathiazole III**	Fumaric acid	n (ball milling)	[28]
**Sulfathiazole III**	Malic acid	n (ball milling)	[28]
**Sulfathiazole III**	Malonic acid	n (ball milling)	[28]
**Sulfathiazole III**	Succinic acid	n (ball milling)	[28]
**Sulfathiazole III**	Glutaric acid	n (ball milling)	[28]
**Sulfathiazole III**	Adipic acid	n (ball milling)	[28]
**Sulfathiazole III**	Pimelic acid	n (ball milling)	[28]
**Sulfathiazole V**	Oxalic acid	n (ball milling)	[28]
**Sulfathiazole V**	Tartaric acid	y	[28]
**Sulfathiazole V**	Citric acid	y	[28]
**Sulfathiazole V**	Fumaric acid	n (ball milling)	[28]
**Sulfathiazole V**	Malic acid	n (ball milling)	[28]
**Sulfathiazole V**	Malonic acid	n (ball milling)	[28]
**Sulfathiazole V**	Succinic acid	n (ball milling)	[28]
**Sulfathiazole V**	Glutaric acid	n (ball milling)	[28]
**Sulfathiazole V**	Adipic acid	n (ball milling)	[28]
**Sulfathiazole V**	Pimelic acid	n (ball milling)	[28]

References

1. Masuda, T.; Yoshihashi, Y.; Yonemochi, E.; Fujii, K.; Uekusa, H.; Terada, K. Cocrystallization and amorphization induced by drug-excipient interaction improves the physical properties of acyclovir. *Int J Pharm* **2012**, *422*, 160-169, doi:10.1016/j.ijpharm.2011.10.046.
2. Pan, Y.; Pang, W.; Lv, J.; Wang, J.; Yang, C.; Guo, W. Solid state characterization of azelnidipine-oxalic acid co-crystal and co-amorphous complexes: The effect of different azelnidipine polymorphs. *J Pharm Biomed Anal* **2017**, *138*, 302-315, doi:10.1016/j.jpba.2017.02.005.
3. Han, Y.; Pan, Y.; Lv, J.; Guo, W.; Wang, J. Powder grinding preparation of co-amorphous β-azelnidipine and maleic acid combination: Molecular interactions and physicochemical properties. *Powder Technol.* **2016**, *291*, 110-120, doi:10.1016/j.powtec.2015.11.068.
4. Ueda, H.; Wu, W.; Lobmann, K.; Grohganz, H.; Mullertz, A.; Rades, T. Application of a Salt Coformer in a Co-Amorphous Drug System Dramatically Enhances the Glass Transition Temperature: A Case Study of the Ternary System Carbamazepine, Citric Acid, and l-Arginine. *Mol Pharm* **2018**, *15*, 2036-2044, doi:10.1021/acs.molpharmaceut.8b00174.
5. Wu, W.; Wang, Y.; Loebmann, K.; Grohganz, H.; Rades, T. Transformations between co-amorphous and co-crystal systems and their influence on the formation and physical stability of co-amorphous systems. *Molecular Pharmaceutics* **2019**, 10.1021/acs.molpharmaceut.8b01229, doi:10.1021/acs.molpharmaceut.8b01229.
6. Wu, W.; Ueda, H.; Lobmann, K.; Rades, T.; Grohganz, H. Organic acids as co-formers for co-amorphous systems - Influence of variation in molar ratio on the physicochemical properties of the co-amorphous systems. *Eur J Pharm Biopharm* **2018**, *131*, 25-32, doi:10.1016/j.ejpb.2018.07.016.
7. Mohammed, A.; Zurek, J.; Madueke, S.; Al-Kassimy, H.; Yaqoob, M.; Houacine, C.; Ferraz, A.; Kalgudi, R.; Zariwala, M.G.; Hawkins, N., et al. Generation of High Dose Inhalable Effervescent Dispersions against Pseudomonas aeruginosa Biofilms. *Pharm. Res.* **2020**, *37*, 150, doi:10.1007/s11095-020-02878-w.
8. Paluch, K.J.; McCabe, T.; Muller-Bunz, H.; Corrigan, O.I.; Healy, A.M.; Tajber, L. Formation and physicochemical properties of crystalline and amorphous salts with different stoichiometries formed between ciprofloxacin and succinic acid. *Mol Pharm* **2013**, *10*, 3640-3654, doi:10.1021/mp400127r.
9. Ali, A.M.; Ali, A.A.; Maghrabi, I.A. Clozapine-carboxylic acid plasticized co-amorphous dispersions: Preparation, characterization and solution stability evaluation. *Acta. Pharm.* **2015**, *65*, 133-146, doi:10.1515/acph-2015-0014.
10. Skieneh, J.M.; Sathisaran, I.; Dalvi, S.V.; Rohani, S. Co-amorphous Form of Curcumin–Folic Acid Dihydrate with Increased Dissolution Rate. *Cryst. Growth Des.* **2017**, *17*, 6273-6280, doi:10.1021/acs.cgd.7b00947.
11. Ainurofiq, A.; Mauludin, R.; Mudhakir, D.; Soewandhi, S.N. A Novel Desloratadine-Benzoic Acid Co-Amorphous Solid: Preparation, Characterization, and Stability Evaluation. *Pharmaceutics* **2018**, *10*, doi:10.3390/pharmaceutics10030085.
12. Zhang, M.; Suo, Z.; Peng, X.; Gan, N.; Zhao, L.; Tang, P.; Wei, X.; Li, H. Microcrystalline cellulose as an effective crystal growth inhibitor for the ternary Ibrutinib formulation. *Carbohydr. Polym.* **2020**, *229*, 115476, doi:10.1016/j.carbpol.2019.115476.
13. Lu, Q.; Zografi, G. Phase behavior of binary and ternary amorphous mixtures containing indomethacin, citric acid, and PVP. *Pharm. Res.* **1998**, *15*, 1202-1206.

14. Yamamoto, K.; Kojima, T.; Karashima, M.; Ikeda, Y. Physicochemical evaluation and developability assessment of co-amorphouses of low soluble drugs and comparison to the co-crystals. *Chemical and Pharmaceutical Bulletin* **2016**, c16-00604.
15. Fung, M.; Be Rzins, K.R.; Suryanarayanan, R. Physical Stability and Dissolution Behavior of Ketoconazole-Organic Acid Coamorphous Systems. *Mol Pharm* **2018**, *15*, 1862-1869, doi:10.1021/acs.molpharmaceut.8b00035.
16. Fung, M.H.; DeVault, M.; Kuwata, K.T.; Suryanarayanan, R. Drug-Excipient Interactions: Effect on Molecular Mobility and Physical Stability of Ketoconazole-Organic Acid Coamorphous Systems. *Mol Pharm* **2018**, *15*, 1052-1061, doi:10.1021/acs.molpharmaceut.7b00932.
17. Wang, J.; Chang, R.; Zhao, Y.; Zhang, J.; Zhang, T.; Fu, Q.; Chang, C.; Zeng, A. Coamorphous Loratadine-Citric Acid System with Enhanced Physical Stability and Bioavailability. *AAPS PharmSciTech* **2017**, *18*, 2541-2550, doi:10.1208/s12249-017-0734-0.
18. Chambers, L.I.; Grohganz, H.; Palmelund, H.; Lobmann, K.; Rades, T.; Musa, O.M.; Steed, J.W. Predictive Identification of Co-formers in Co-amorphous Systems. *Eur J Pharm Sci* **2020**, 10.1016/j.ejps.2020.105636, 105636, doi:10.1016/j.ejps.2020.105636.
19. An, J.H.; Lim, C.; Kiyonga, A.N.; Chung, I.H.; Lee, I.K.; Mo, K.; Park, M.; Youn, W.; Choi, W.R.; Suh, Y.G., et al. Co-Amorphous Screening for the Solubility Enhancement of Poorly Water-Soluble Mirabegron and Investigation of Their Intermolecular Interactions and Dissolution Behaviors. *Pharmaceutics* **2018**, *10*, doi:10.3390/pharmaceutics10030149.
20. Ueda, H.; Muranushi, N.; Sakuma, S.; Ida, Y.; Endoh, T.; Kadota, K.; Tozuka, Y. A Strategy for Co-former Selection to Design Stable Co-amorphous Formations Based on Physicochemical Properties of Non-steroidal Inflammatory Drugs. *Pharm. Res.* **2016**, *33*, 1018-1029, doi:10.1007/s11095-015-1848-2.
21. Maher, E.M.; Ali, A.M.; Salem, H.F.; Abdelrahman, A.A. In vitro/in vivo evaluation of an optimized fast dissolving oral film containing olanzapine co-amorphous dispersion with selected carboxylic acids. *Drug Deliv* **2016**, *23*, 3088-3100, doi:10.3109/10717544.2016.1153746.
22. Hoppu, P.; Hietala, S.; Schantz, S.; Juppo, A.M. Rheology and molecular mobility of amorphous blends of citric acid and paracetamol. *Eur J Pharm Biopharm* **2009**, *71*, 55-63, doi:10.1016/j.ejpb.2008.06.029.
23. Hoppu, P.; Jouppila, K.; Rantanen, J.; Schantz, S.; Juppo, A.M. Characterisation of blends of paracetamol and citric acid. *J Pharm Pharmacol* **2007**, *59*, 373-381, doi:10.1211/jpp.59.3.0006.
24. Ahuja, N.; Katare, O.P.; Singh, B. Studies on dissolution enhancement and mathematical modeling of drug release of a poorly water-soluble drug using water-soluble carriers. *Eur J Pharm Biopharm* **2007**, *65*, 26-38, doi:10.1016/j.ejpb.2006.07.007.
25. Hirakawa, Y.; Ueda, H.; Takata, Y.; Minamihata, K.; Wakabayashi, R.; Kamiya, N.; Goto, M. Co-amorphous formation of piroxicam-citric acid to generate supersaturation and improve skin permeation. *Eur J Pharm Sci* **2021**, *158*, 105667, doi:10.1016/j.ejps.2020.105667.
26. Gniado, K.; Lobmann, K.; Rades, T.; Erxleben, A. The influence of co-formers on the dissolution rates of co-amorphous sulfamerazine/excipient systems. *Int J Pharm* **2016**, *504*, 20-26, doi:10.1016/j.ijpharm.2016.03.023.
27. Macfhionnghaile, P.; Hu, Y.; Gniado, K.; Curran, S.; McArdle, P.; Erxleben, A. Effects of ball-milling and cryomilling on sulfamerazine polymorphs: a quantitative study. *J. Pharm. Sci.* **2014**, *103*, 1766-1778, doi:10.1002/jps.23978.
28. Hu, Y.; Gniado, K.; Erxleben, A.; McArdle, P. Mechanochemical Reaction of Sulfathiazole with Carboxylic Acids: Formation of a Cocrystal, a Salt, and Coamorphous Solids. *Cryst. Growth Des.* **2014**, *14*, 803-813, doi:10.1021/cg401673z.

Table S4. Overview of screened CAMS and non-CAMS with various (other) co-formers. (y: yes. n: no. -: unknown).

Drug	Coformer	CAMS	Reference
**6-Mercaptopurine**	Sodium taurocholate	y	[1]
**Acyclovir**	Sodium taurocholate	y	[1]
**Allopurinol**	Sodium taurocholate	y	[1]
**Amoxicillin**	Sodium taurocholate	y	[1]
**Atenolol**	Urea	y	[2]
**Atorvastatin calcium**	Nicotinamide	y	[3]
**Azithromycin**	N-Acetylcysteine	y	[4]
**Benzamidine**	Sodium taurocholate	y	[1]
**Carbamazepine**	Sodium taurocholate	y	[1]
**Carbamazepine**	Tannic acid	y	[5]
**Carbamazepine**	Saccharin	y	[6]
**Carbamazepine**	Nicotinamide	y	[6]
**Ciprofloxacin**	Sodium taurocholate	y	[1]
**Ciprofloxacin**	N-Acetylcysteine	n (spray drying)	[4]
**Curcumin**	Piperazine	y	[7]

**Diflusinal**	Sodium taurocholate	y	[1]
**Glibenclamide**	Quercetin	y	[8]
**Hydrochlorothiazide**	Sodium lauryl sulfate	n (ball milling)	[9]
**Ibrutinib**	Oxalic acid	y	[10]
**Ibrutinib**	Saccharin	y	[11]
**Ibuprofen**	Nicotinamide	y	[12]
**Ibuprofen**	Sodium taurocholate	y	[1]
**Indomethacin**	Sodium taurocholate	y	[1]
**Indomethacin**	Tannic acid	Y	[5]
**Lurasidone hydrochloride**	Saccharin	y	[13]
**Mebendazole**	Glutamic acid-Arginine	n (ball milling)	[14]
**Mebendazole**	Arginine-Glutamic acid	n (ball milling)	[14]
**Mebendazole**	Aspartame	y	[15]
**Mebendazole**	Tryptophan-Phenylalanine	y	[16]
**Mebendazole**	Phenylalanine-Tryptophan	y	[16]
**Mebendazole**	Aspartic acid-Tyrosine	y	[16]
**Mebendazole**	Histidine-Glycine	y	[16]
**Mebendazole**	Proline-Tryptophan	y	[16]
**Mebendazole**	Glutamic acid-Arginine (crystalline salt)	y	[14]
**Mebendazole**	Glutamic acid-Arginine (amorphous salt)	y	[14]
**Mebendazole**	4,4′-Bipryidine	n (ball milling)	[17]
**Mebendazole**	Imidazole	n (ball milling)	[17]
**Mebendazole**	Isonicotinamide	n (ball milling)	[17]
**Mebendazole**	Nicotinamide	n (ball milling)	[17]
**Mebendazole**	Phenazine	n (ball milling)	[17]
**Mebendazole**	Piperazine	n (ball milling)	[17]
**Mebendazole**	Pyrogallol	y	[17]
**Mebendazole**	Salicylic acid	y	[17]
**Mebendazole**	Urea	n (ball milling)	[17]
**Mefenamic acid**	Sodium taurocholate	y	[1]
**Naproxen**	Sodium taurocholate	y	[1]
**Naproxen**	Meglumine	y	[18]
**Nifedipine**	Sodium taurocholate	y	[1]
**Norfloxacin**	Sodium taurocholate	y	[1]
**Olanzapine**	Saccharin	y	[19]
**Paracetamol**	Sodium taurocholate	y	[1]
**Piroxicam**	Aspartame	y	[15]
**Probucol**	Maltitol	n (melt-quench)	[20]
**Quetiapine**	Nicotinamide	n (freeze drying)	[21]
**Quinine**	Sodium taurocholate	y	[1]
**Repaglinide**	Saccharin	y	[22]
**Rofecoxib**	Mannitol	-	[23]
**Rofecoxib**	Sorbitol	-	[23]
**Rofecoxib**	Urea	-	[23]
**Rofecoxib**	Nicotinamide	-	[23]
**ROY**	Pyrogallol	y	[24]
**Sulfamerazine**	Sodium taurocholate	y	[1]
**Sulfamerazine**	Mannitol	n (melt-quench)	[25]
**Sulfamerazine**	Saccharin	n (melt-quench)	[25]
**Sulfamerazine**	Sorbitol	n (melt-quench)	[25]
**Sulfathiazole**	Sodium taurocholate	y	[1]

**Tadalafil**	Aspartame	y	[15]
**Tobramycin**	N-Acetylcysteine	y	[4]
**Ursolic acid**	Piperine	y	[26]
**Valsartan**	Vanillin	y	[27]

References

1. Gniado, K.; MacFhionnghaile, P.; McArdle, P.; Erxleben, A. The natural bile acid surfactant sodium taurocholate (NaTC) as a coformer in coamorphous systems: Enhanced physical stability and dissolution behavior of coamorphous drug-NaTc systems. *Int J Pharm* **2018**, *535*, 132-139, doi:10.1016/j.ijpharm.2017.10.049.
2. Hirakawa, Y.; Ueda, H.; Miyano, T.; Kamiya, N.; Goto, M. New insight into transdermal drug delivery with supersaturated formulation based on co-amorphous system. *Int J Pharm* **2019**, *569*, 118582, doi:10.1016/j.ijpharm.2019.118582.
3. Shayanfar, A.; Jouyban, A. Drug–Drug Coamorphous Systems: Characterization and Physicochemical Properties of Coamorphous Atorvastatin with Carvedilol and Glibenclamide. *Journal of Pharmaceutical Innovation* **2013**, *8*, 218-228, doi:10.1007/s12247-013-9162-1.
4. Lababidi, N.; Ofosu Kissi, E.; Elgaher, W.A.M.; Sigal, V.; Haupenthal, J.; Schwarz, B.C.; Hirsch, A.K.H.; Rades, T.; Schneider, M. Spray-drying of inhalable, multifunctional formulations for the treatment of biofilms formed in cystic fibrosis. *J Control Release* **2019**, *314*, 62-71, doi:10.1016/j.jconrel.2019.10.038.
5. Fael, H.; Demirel, A.L. Tannic acid as a co-former in co-amorphous systems: Enhancing their physical stability, solubility and dissolution behavior. *Int J Pharm* **2020**, *581*, 119284, doi:10.1016/j.ijpharm.2020.119284.
6. Wu, W.; Wang, Y.; Loebmann, K.; Grohganz, H.; Rades, T. Transformations between co-amorphous and co-crystal systems and their influence on the formation and physical stability of co-amorphous systems. *Molecular Pharmaceutics* **2019**, 10.1021/acs.molpharmaceut.8b01229, doi:10.1021/acs.molpharmaceut.8b01229.
7. Pang, W.; Lv, J.; Du, S.; Wang, J.; Wang, J.; Zeng, Y. Preparation of Curcumin-Piperazine Coamorphous Phase and Fluorescence Spectroscopic and Density Functional Theory Simulation Studies on the Interaction with Bovine Serum Albumin. *Mol Pharm* **2017**, *14*, 3013-3024, doi:10.1021/acs.molpharmaceut.7b00217.
8. Sormunen, H.; Ruponen, M.; Laitinen, R. The effect of co-amorphization of glibenclamide on its dissolution properties and permeability through an MDCKII-MDR1 cell layer. *Int J Pharm* **2019**, *570*, 118653, doi:10.1016/j.ijpharm.2019.118653.
9. Ruponen, M.; Rusanen, H.; Laitinen, R. Dissolution and Permeability Properties of Co-Amorphous Formulations of Hydrochlorothiazide. *J. Pharm. Sci.* **2020**, *109*, 2252-2261, doi:10.1016/j.xphs.2020.04.008.
10. Zhang, M.; Suo, Z.; Peng, X.; Gan, N.; Zhao, L.; Tang, P.; Wei, X.; Li, H. Microcrystalline cellulose as an effective crystal growth inhibitor for the ternary Ibrutinib formulation. *Carbohydr. Polym.* **2020**, *229*, 115476, doi:10.1016/j.carbpol.2019.115476.
11. Shi, X.; Song, S.; Ding, Z.; Fan, B.; Huang, W.; Xu, T. Improving the Solubility, Dissolution, and Bioavailability of Ibrutinib by Preparing It in a Coamorphous State With Saccharin. *J. Pharm. Sci.* **2019**, *108*, 3020-3028, doi:10.1016/j.xphs.2019.04.031.
12. Bi, Y.; Xiao, D.; Ren, S.; Bi, S.; Wang, J.; Li, F. The Binary System of Ibuprofen-Nicotinamide Under Nanoscale Confinement: From Cocrystal to Coamorphous State. *J. Pharm. Sci.* **2017**, *106*, 3150-3155, doi:10.1016/j.xphs.2017.06.005.
13. Qian, S.; Heng, W.; Wei, Y.; Zhang, J.; Gao, Y. Coamorphous Lurasidone Hydrochloride–Saccharin with Charge-Assisted Hydrogen Bonding Interaction Shows Improved Physical Stability and Enhanced Dissolution with pH-Independent Solubility Behavior. *Cryst. Growth Des.* **2015**, *15*, 2920-2928, doi:10.1021/acs.cgd.5b00349.
14. Wu, W.; Grohganz, H.; Rades, T.; Lobmann, K. Comparison of co-former performance in co-amorphous formulations: single amino acids, amino acid physical mixtures, amino acid salts and dipeptides as co-formers. *Eur J Pharm Sci* **2020**, 10.1016/j.ejps.2020.105582, 105582, doi:10.1016/j.ejps.2020.105582.
15. Wu, W.; Lobmann, K.; Schnitzkewitz, J.; Knuhtsen, A.; Pedersen, D.S.; Grohganz, H.; Rades, T. Aspartame as a co-former in co-amorphous systems. *Int J Pharm* **2018**, *549*, 380-387, doi:10.1016/j.ijpharm.2018.07.063.
16. Wu, W.; Lobmann, K.; Schnitzkewitz, J.; Knuhtsen, A.; Pedersen, D.S.; Rades, T.; Grohganz, H. Dipeptides as co-formers in co-amorphous systems. *Eur J Pharm Biopharm* **2019**, *134*, 68-76, doi:10.1016/j.ejpb.2018.11.016.
17. Chambers, L.I.; Grohganz, H.; Palmelund, H.; Lobmann, K.; Rades, T.; Musa, O.M.; Steed, J.W. Predictive Identification of Co-formers in Co-amorphous Systems. *Eur J Pharm Sci* **2020**, 10.1016/j.ejps.2020.105636, 105636, doi:10.1016/j.ejps.2020.105636.
18. Liu, X.; Zhou, L.; Zhang, F. Reactive Melt Extrusion To Improve the Dissolution Performance and Physical Stability of Naproxen Amorphous Solid Dispersions. *Mol Pharm* **2017**, *14*, 658-673, doi:10.1021/acs.molpharmaceut.6b00960.
19. da Costa, N.F.; Fernandes, A.I.; Pinto, J.F. Measurement of the amorphous fraction of olanzapine incorporated in a co-amorphous formulation. *Int J Pharm* **2020**, *588*, 119716, doi:10.1016/j.ijpharm.2020.119716.
20. Mizoguchi, R.; Waraya, H.; Hirakura, Y. Application of Co-Amorphous Technology for Improving the Physicochemical Properties of Amorphous Formulations. *Mol Pharm* **2019**, *16*, 2142-2152, doi:10.1021/acs.molpharmaceut.9b00105.
21. Ali, A.M.; Al-Remawi, M.M. Freeze Dried Quetiapine-Nicotinamide Binary Solid Dispersions: A New Strategy for Improving Physicochemical Properties and Ex Vivo Diffusion. *J Pharm (Cairo)* **2016**, *2016*, 2126056, doi:10.1155/2016/2126056.

22. Gao, Y.; Liao, J.; Qi, X.; Zhang, J. Coamorphous repaglinide-saccharin with enhanced dissolution. *Int J Pharm* **2013**, *450*, 290-295, doi:10.1016/j.ijpharm.2013.04.032.
23. Ahuja, N.; Katare, O.P.; Singh, B. Studies on dissolution enhancement and mathematical modeling of drug release of a poorly water-soluble drug using water-soluble carriers. *Eur J Pharm Biopharm* **2007**, *65*, 26-38, doi:10.1016/j.ejpb.2006.07.007.
24. Corner, P.A.; Harburn, J.J.; Steed, J.W.; McCabe, J.F.; Berry, D.J. Stabilisation of an amorphous form of ROY through a predicted co-former interaction. *Chem. Commun. (Camb.)* **2016**, *52*, 6537-6540, doi:10.1039/c6cc02949c.
25. Macfhionnghaile, P.; Hu, Y.; Gniado, K.; Curran, S.; McArdle, P.; Erxleben, A. Effects of ball-milling and cryomilling on sulfamerazine polymorphs: a quantitative study. *J. Pharm. Sci.* **2014**, *103*, 1766-1778, doi:10.1002/jps.23978.
26. Yu, D.; Kan, Z.; Shan, F.; Zang, J.; Zhou, J. Triple Strategies to Improve Oral Bioavailability by Fabricating Coamorphous Forms of Ursolic Acid with Piperine: Enhancing Water-Solubility, Permeability, and Inhibiting Cytochrome P450 Isozymes. *Mol Pharm* **2020**, *17*, 4443-4462, doi:10.1021/acs.molpharmaceut.0c00443.
27. Hashim Ali, K.; Mohsin Ansari, M.; Ali Shah, F.; Ud Din, F.; Abdul Basit, M.; Kim, J.K.; Zeb, A. Enhanced dissolution of valsartan-vanillin binary co-amorphous system loaded in mesoporous silica particles. *J Microencapsul* **2019**, *36*, 10-20, doi:10.1080/02652048.2019.1579265.
